# Essential role of *cis*-encoded mature NS3 in the genome packaging of classical swine fever virus

**DOI:** 10.1128/jvi.01209-24

**Published:** 2024-12-26

**Authors:** Benjamin Lamp, Sandra Barth, Carina Reuscher, Sebastian Affeldt, Angelika Cechini, Anette Netsch, Irmin Lobedank, Till Rümenapf

**Affiliations:** 1 Institute of Virology, Justus-Liebig-Universität Gießen 9175 , Giessen, Hesse, Germany; 2 Institute of Virology, Department for Pathobiology, University of Veterinary Medicine 27260 , Vienna, Austria; University of Kentucky College of Medicine, Lexington, Kentucky, USA

**Keywords:** classical swine fever virus, CSFV, pestivirus, NS3, virus assembly, genome packaging, coreless virus, cytopathogenicity, *trans*-complementation, morphogenesis

## Abstract

Classical swine fever virus (CSFV) is a member of the genus *Pestivirus* within the family *Flaviviridae*. The enveloped particles contain a plus-stranded RNA genome encoding a single large polyprotein. The processing of this polyprotein undergoes dynamic changes throughout the infection cycle. The release of mature NS3 from the polyprotein is mediated and regulated by the NS2 autoprotease and a cellular co-factor, restricting efficient cleavage to the early phases of infection. NS3 is a multifunctional viral enzyme exhibiting helicase, NTPase, and protease activities pivotal for viral replication. Hence, the release of mature NS3 fuels replication, whereas unprocessed NS2-3 precursors are vital for progeny virus production in later phases of infection. Thus far, no packaging signals have been identified for pestivirus RNA. To explore the prerequisites for particle assembly, *trans*-packaging experiments were conducted using CSFV subgenomes and coreless CSFV strains. Intriguingly, we discovered a significant role of mature NS3 in genome packaging, effective only when the protein is encoded by the RNA molecule itself. This finding was reinforced by employing artificially engineered CSFV strains with duplicated NS3 genes, separating uncleavable NS2-3 precursors from mature NS3 molecules. The model for NS2-3/NS3 functions in genome packaging of pestiviruses appears to be much more complicated than anticipated, involving distinct functions of the mature NS3 and its precursor molecule NS2-3. Moreover, the reliance of genome packaging on *cis*-encoded NS3 may act as a downstream quality control mechanism, averting the packaging of defective genomes and coordinating the encapsidation of RNA molecules before membrane acquisition.

**IMPORTANCE:**

Pestiviruses are economically significant pathogens in livestock. Although genome organization and non-structural protein functions resemble those of other *Flaviviridae* genera, distinct differences can be observed. Previous studies showed that coreless CSFV strains can produce coreless virions mediated by single compensatory mutations in NS3. In this study, we could show that only RNA molecules encoding these mutations in the mature NS3 are packaged in the absence of the core protein. Unlike this selectivity, a pool of structural proteins in the host cell was readily available for packaging all CSFV genomes. Similarly, the NS2-3-4A precursor molecules required for packaging could also be provided in *trans*. Consequently, genome packaging in pestiviruses is governed by *cis*-encoded mature NS3. Reliance on *cis*-acting proteins restricts the acceptance of defective genomes and establishes packaging specificity regardless of RNA sequence-specific packaging signals. Understanding the role of NS3 in pestiviral genome packaging might uncover new targets for antiviral therapies.

## INTRODUCTION

The classical swine fever virus (CSFV) belongs to the species *Pestivirus C* grouped within the genus *Pestivirus* in the family *Flaviviridae* (1). Pestiviruses are renowned in veterinary circles for causing economically significant diseases in cloven-hoofed animals. Recent years have seen the discovery of numerous new pestivirus species, prompting a taxonomic re-classification. Now, the species are categorized as pestiviruses A–G, replacing the traditional names, which were based on the host species and the type of disease (2). Newly found pestiviruses have expanded the host range of the genus beyond ungulates to include rodents (3), bats (3), pangolins (4), and even whales (5). Other genera within the *Flaviviridae* family, such as *Flavivirus*, *Pegivirus*, and *Hepacivirus*, harbor significant human pathogens. All *Flaviviridae* share similarities in their genome organization, non-structural protein enzyme activities, and viral lifecycle (1). However, there are also major differences in polyprotein processing, the structural protein region, and morphogenesis.

Pestiviruses are small, enveloped agents with single-stranded RNA genomes of positive polarity. The genome encodes a single large polyprotein, which is co- and post-translationally processed into mature proteins by cellular and viral proteases (1). Although structural proteins exhibit considerable inter-genera diversity among the *Flaviviridae*, the non-structural ones show more similarities in terms of processing, domain architecture, and three-dimensional structure. At the heart of the viral lifecycle sits NS3, containing a chymotrypsin-like serine protease domain, which cleaves the viral polypeptides at *cis* (NS3-4) and *trans* (NS4-5) sites (6), aided by NS4A (in genus *Pestivirus*, *Hepacivirus*, and *Pegivirus* [7]) or NS2B (in genus *Flavivirus* [8]) as a protease co-factor. Beyond its N-terminal protease domain, NS3 possesses conserved helicase and NTPase domains, both indispensable for genome replication (9). The DExH/D-box helicase (superfamily 2) is located in the molecule’s C-terminus and exhibits significant motif conservation (10). Various *in vitro* models have elucidated the unwinding activities of the NS3 helicases of pestiviruses (11), different flaviviruses (12), and the hepatitis C virus (HCV) (13).

In pestiviruses, a cellular protein known as DNAJC14 or JIV (J-domain protein interacting with viral protein) plays a crucial role as a co-factor for the NS2 autoprotease, enabling the processing of NS2-3 and thus controlling the release of mature NS3 (14). Inhibition of NS2-3 processing due to JIV deficiency acts as a molecular switch that determines the transition from initial accelerated replication to subsequent particle morphogenesis and reduced replication. Although mature NS3 is indispensable for genome replication (15), uncleaved NS2-3 is essential for particle assembly (16, 17). Recent studies employing synthetic pestiviruses, wherein NS2 and NS3 were separated by an IRES element, demonstrated that two mutations (E_1576_V in NS2 and V_1721_A in NS3) are sufficient to allow NS2-3 independent morphogenesis (18, 19). This finding suggests a significant contribution of mature NS3 to genome packaging in pestiviruses or at least an uncomplicated restructuring of the process. The coupling of RNA replication to cellular metabolism enables pestiviruses to replicate in cultured cells in a non-cytopathogenic (ncp) manner. However, cytopathogenic (cp) variants of pestiviruses emerge following recombination with cellular mRNAs or the introduction of activating mutations within the NS2 autoprotease gene. Hallmark of such cp pestiviruses is an efficient maturation of NS3 independent of cellular DNAJC14 levels (20). The elevated NS3 levels of mutant genomes correlate with accelerated processing of NS4-5 precursor molecules, enhanced RNA replication, increased viral protein abundance, and ultimately apoptosis-mediated death of infected host cells (21, 22).

A commonly used safety measure in laboratory work with *Flaviviridae* involves deleting the capsid or core protein gene sequence from the genome, effectively preventing the formation of infectious particles (23, 24). However, previous studies revealed that single point mutations within domain 3 of the NS3 helicase of CSFV can compensate for the absence of core, enabling viral morphogenesis even in the complete absence of a capsid protein (25). In this study, we utilized coreless CSFVs to explore the molecular mechanisms of morphogenesis, employing well-established RNA replicon *trans*-packaging models (26) and newly engineered CSFV genomes with sequence duplications of the NS3 region (27). Through this, we pinpointed a unique *cis* function to the mature NS3 molecule in CSFV genome packaging. The activity of mature NS3 was not just evident in *trans-*packaging assays using defective interfering genomes (DIs) but also documented for autonomously particle-forming cp variants of CSFV, shedding light on the intricate mechanisms governing viral RNA packaging specificity in CSFV.

## MATERIALS AND METHODS

### Cells and viruses

SK-6 (swine kidney 6 [28]) cells were cultivated in Dulbecco’s modified Eagle’s medium (DMEM) supplemented with 10% fetal calf serum (FCS; Gold Bio&Sell, Feucht, Germany) at 37°C with 5% CO_2_. Recombinant CSFV strains and pestivirus (sub-)genomes were generated as described earlier (27). Throughout this study, nucleotide and amino acid numbering of CSFV refers to the sequence of the parental ncp CSFV strain Alfort-Tübingen (Genbank J04358.2 [29]). The genomic sequence of Linda pestivirus (GenBank: KY436034.1) and the sequence of the bovine diarrhea virus 1 (BVDV-1) strain CP7 (U63479.1) were used as references for Linda pestivirus and the BVDV-1 subgenome DI9.

### Generation of CSFV cDNA clones

**Fig 1:**
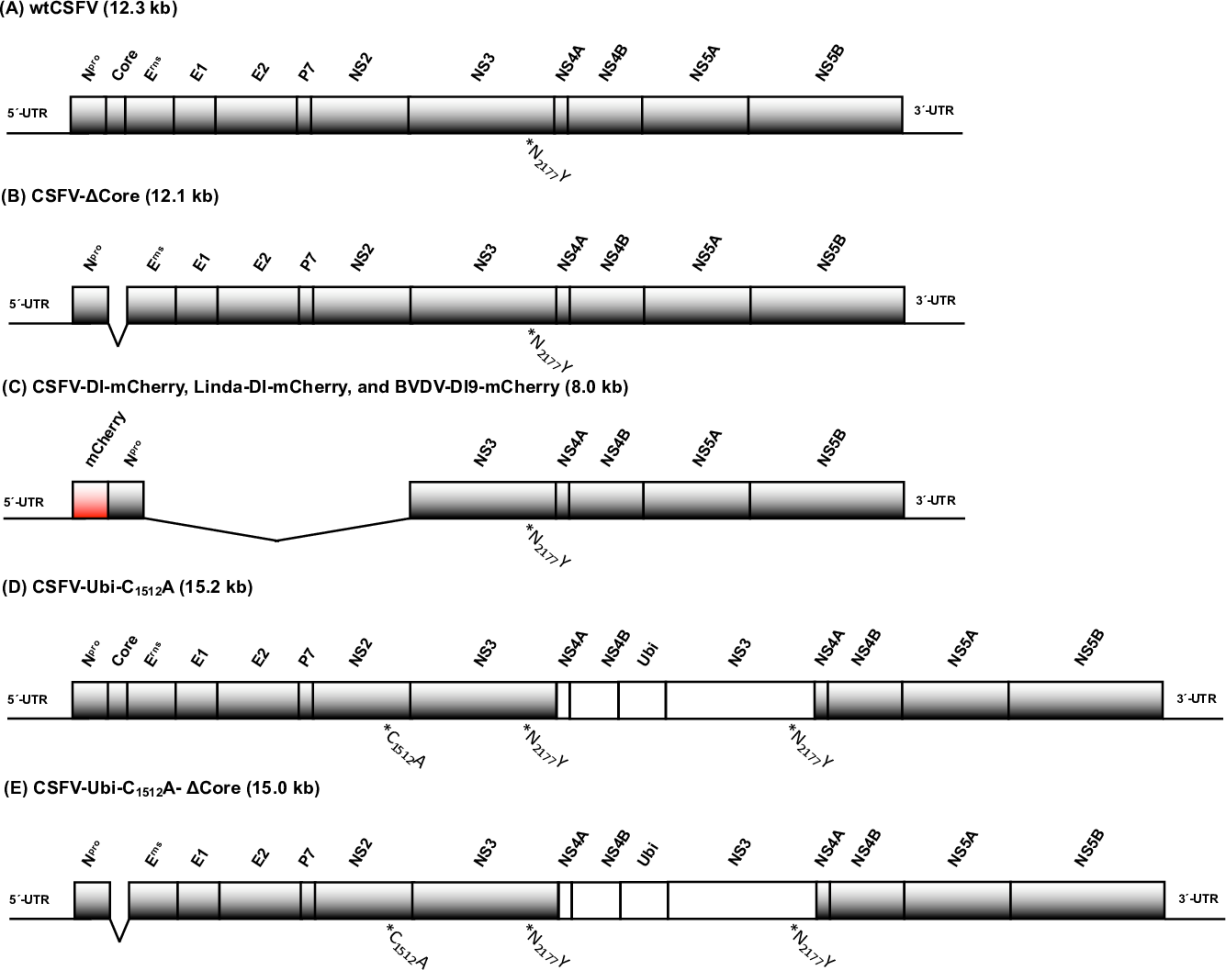
Recombinant viral genomes used in this study. (**A**) The genome organization of wtCSFV is illustrated. Captions encompass the 5′-UTR, the ORF including N^pro^, core, E^rns^, E1, E2, P7, NS2, NS3, NS4A, NS4B, NS5A, NS5B, and the 3′-UTR. A star highlights the mutation N_2177_Y, which can compensate for the lack of core protein. (**B**) Genome organization of a coreless CSFV (CSFVΔcore) including corresponding captions. (**C**) Genome organization of pestiviral replicons. A reporter gene (mCherry) was positioned after amino acid 15 of N^pro^, whereas the core to NS2 genes is deleted in the CSFV-DI-mCherry and the Linda-DI-mCherry. The BVDV-1 DI9 was cloned many years ago from a field case of mucosal disease, and we only had to insert a reporter gene to generate BVDV-DI-mCherry. (**D**) Genome organization of a cytopathogenic CSFV (CSFV-Ubi) with duplicated NS3–NS4B genes. The active cysteine (C_1512_) was exchanged against an alanine to prevent NS2-3 maturation. The mutation N_2177_Y, compensating for the lack of core protein, was introduced into either NS2-3 or NS3 genes. (**E**) Genome organization of a coreless cytopathogenic CSFV. Alongside the designation of the different virus genomes, the genome size is specified in parentheses.

Figure 1 shows the genome organization of all plasmid or bacterial artificial chromosome (BAC)-derived CSFV genomes and pestiviral subgenomes used in this study (Fig. 1). Table 1 contains further details on the individual clones, including a list of their respective mutations and a brief description of their properties (Table 1). Previously, we introduced cDNA plasmid clones of both, the wild-type CSFV (wtCSFV, Fig. 1A) and the coreless CSFV (CSFV-ΔCore, Fig. 1B), along with mutations within the helicase domain of NS3 compensating for core deficiency (CSFV-N_2177_Y or CSFV-ΔCore-N_2177_Y) (25). Replicative CSFV subgenomes (CSFV-DI, Fig. 1C) (21) and cp CSFV strains featuring duplicated NS3–NS4B regions (CSFV-Ubi, Fig. 1D) have also been described earlier (27). The BVDV-1 subgenome DI9 and its plasmid clone pDI9 are well-known (30, 31). The generation of CSFV strains and the introduction of mutations were performed by similar strategies as detailed in (27). Briefly, the methodology used involved PCR-based mutagenesis (Q5; NEB, Ipswich, MA, USA) and seamless DNA assembly reactions (NEBuilder HiFi DNA Assembly; NEB). Details of the oligonucleotide sequences used in this study can be found in Table 2. Details on PCR conditions and general standard cloning techniques are available upon request from the corresponding author. To ensure accuracy, all mutagenized DNA plasmids and BACs underwent thorough verification via pore nucleotide sequencing conducted by a commercial service provider (Full plasmid sequencing; Microsynth AG, Balgach, Switzerland).

**TABLE 1:** CSFV and pestiviral (sub-)genomes used in this study

Designation	Scheme of the genome	Mutations, modifications (Δ, I, M), and their genome position (nt)^*a*^	Virus properties
wtCSFV	Fig. 1A	^*b*^-	Infectious wild-type CSFV clone (29).
CSFV-Δcore	Fig. 1B	Δ nt _879_C-_1112_T	Coreless CSFV clone without compensatory NS3 mutations. Replication competent subgenome without infectious progeny virus production (25).
CSFV-N_2177_Y	Fig. 1A	M nt _6901_AAT to _6901_TAC	Infectious wild-type CSFV with compensatory NS3 mutation. Replication of competent genome with infectious progeny virus production (25).
CSFV-Δcore-N_2177_Y	Fig. 1B	Δ nt _879_C-_1112_T M nt _6901_AAT to _6901_TAC	Coreless CSFV clone with compensatory NS3 mutations. Replication competent subgenome with infectious progeny virus production (25).
CSFV-DI	Fig. 1C	Δ nt _877_C-_5139_A	Replication competent subgenome lacking the core to NS2 coding region. No infectious progeny virus production without helper virus (21).
CSFV-DI-mCherry	Fig. 1C	Δ nt _877_C-_5139_A I nt _417_A mCherry gene	Replication competent subgenome lacking core to NS2 coding region labeled with mCherry at amino acid position 15. No infectious progeny virus production without helper virus.
CSFV-DI-N_2177_Y	Fig. 1C	Δ nt _877_C-_5139_A M nt _6901_AAT to _6901_TAC	Replication competent subgenome lacking the core to NS2 coding region including the compensatory NS3 mutation. No infectious progeny virus production without helper virus.
CSFV-DI-N_2177_Y- mCherry	Fig. 1C	Δ nt _877_C-_5139_A M nt _6901_AAT to _6901_TAC I nt _417_A mCherry gene	Replication competent subgenome lacking the core to NS2 coding region labeled with mCherry at amino acid position 15. Containing the compensatory NS3 mutation. No infectious progeny virus production without helper virus.
Linda-DI-mCherry	Fig. 1C	Δ nt _928_T-_5205_G I nt _426_T mCherry gene	Replication competent Linda pestivirus subgenome lacking core to NS2 coding region labeled with mCherry at amino acid position 15. No infectious progeny virus production without helper virus. Not packaged by CSFV.
BVDV-DI9-mCherry	Fig. 1C	Δ nt _873_T-_5162_G I nt _413_A mCherry gene	Replication competent BVDV-1 subgenome lacking core to NS2 coding region labeled with mCherry at amino acid position 15. No infectious progeny virus production without helper virus. Inefficiently packaged by a CSFV helper virus.
CSFV-Ubi-C_1512_A	Fig. 1D	I nt _7776_A ubiquitin gene I _1540_G-_7776_A duplication of NS3 to NS4B M _4906_TGC to _4906_GCC	Infectious cp CSFV with duplicated NS3-4B coding regions behind a ubiquitin processing signal. Non-cleavable NS2-3. Replication competent genome with infectious progeny virus production (27).
CSFV-Ubi- C_1512_A-Δcore	Fig. 1E	I nt _7776_A ubiquitin gene I _1540_G-_7776_A duplication of NS3 to NS4B M _4906_TGC to _4906_GCC Δ nt _879_C-_1112_T	Coreless cp CSFV clone without compensatory NS3 mutation. Replication competent genome including a duplicated NS3-4B coding regions behind a ubiquitin processing signal. Non-cleavable NS2-3. No infectious progeny virus production.
CSFV-Ubi- C_1512_A-Δcore-NS2-3N_2177_Y	Fig. 1E	I nt _7776_A ubiquitin gene insertion I _1540_G-_7776_A duplication of NS3 to NS4B Δ nt _879_C-_1112_T M nt _6901_AAT to _6901_TAC within NS2-3	Coreless cp CSFV clone with compensatory NS3 mutation within the uncleavable NS2-3 moiety. Replication competent genome including duplicated NS3-4B coding regions behind a ubiquitin processing signal. No infectious progeny virus production.
CSFV-Ubi- C_1512_A-Δcore-NS3N_2177_Y	Fig. 1E	I nt _7776_A ubiquitin gene I _1540_G-_7776_A duplication of NS3 to NS4B Δ nt _879_C-_1112_T M nt _6901_AAT to _6901_TAC within the duplicated mature NS3	Coreless cp CSFV clone with compensatory NS3 mutation within the duplicated mature NS3. Non-cleavable NS2-3. Replication competent genome including a duplicated NS3-4B coding regions behind a ubiquitin processing signal. Infectious progeny virus production.

^*a*^ Δ denotes deletion, I insertion, M mutation, and nt means nucleotide.

^*b*^ The “-” simply means no mutations.

**TABLE 2:** Oligonucleotides used in this study

Designation	Sequence (5′ to 3′)
Fwd_mCherry_5′_CN^pro^_5′	CAAAACAAGCAAACAAAGATCTGTGAGCAAGGGCGAGGAGGAT
Rev_mCherry_3′_CN^pro_^5′	CACTCCCACTGGTTTACGCGTCTTGTACAGCTCGTCCATGC
Fwd_CN^pro^_5′	CACTCCCACTGGTTTACGCGTC
Rev_CN^pro^_5′	TTGTTTGCTTGTTTTGTATAATAATTC
Fwd_CN_2177_Y	TACATAATGGCCAGGACCGACCAC
Rev_CN_2177_Y	CCTGGCCATTATGTATTTTACTGCCATCGGCAGCTC
Fwd_mCherry_5′_LN^pro^_5′	AACAACACAAAGAAAGTGAGCAAGGGCGAGGAGGATAAC
Rev_mCherry_3′_LN^pro^_5′	CTCATTATTATTTTTCTTGTACAGCTCGTCCATGCC
Fwd_LN^pro^_5′	AAAAATAATAATGAGGAGGAAGCTGAG
Rev_LN^pro^_5′	TTTCTTTGTGTTGTTGAGAATTTTAAAC
Fwd_mCherry_5′_BN^pro^_5′	GAACTTTTATACAAAGTGAGCAAGGGCGAGGAGGATAAC
Rev_mCherry_3′_BN^pro^_5′	TTTTTGTTTGTATGTCTTGTACAGCTCGTCCATGCC
Fwd_BN^pro^_5′	ACATACAAACAAAAACCCGCTG
Rev_BNpro_5′	TTTGTATAAAAGTTCATTTGTG
Fwd_betalac_GA	GAATGAAGCCATACCAAACGAC
Rev_betalac_GA	GTCGTTTGGTATGGCTTCATTC
Fwd_SPPcore_3′	GGTAACCAGTTGCTCAGAAAAAGCCCTGTTGGCTTGGGCGGTG
Rev_SPPcore_3′	GAGCAACTGGTTACCCATAATGGACAGTTG
Fwd_CAT_GA	GTACTGTTGTAATTCATTAAGCATTCTGCCGAC
Rev_CAT_GA	GTCGGCAGAATGCTTAATGAATTACAACAGTAC
Fwd_NS2_ C_1512_A	GGACCACCAGTGGTCGCCGGTATGACCCTAGCCGATTTC
Rev_ NS2_ C_1512_A	GCGACCACTGGTGGTCCAAAACGCCCAC

A CSFV-DI subgenome with the fluorescent marker gene mCherry at the 5′-end of the ORF was generated here (CSFV-DI-mCherry, Fig. 1C). This insertion was introduced using oligonucleotides Fwd_mCherry_5′_CNpro_5′ and Rev_mCherry_3′_CNpro_5′, along with Fwd_CNpro_5′ and Rev_CNpro_5′, employing PCR amplification and homologous recombination. The NS3 mutation N_2177_Y, compensating for the absence of core protein, was introduced using oligonucleotides Fwd_CN_2177_Y and Rev_CN_2177_Y in the CSFV plasmid clones. A second assembly site located within the beta-lactamase gene (Fwd_betalac and Rev_betalac) was employed to generate suitable PCR products for homologous recombination. The mCherry gene was inserted in a Linda virus DI and the BVDV DI9 using the same recombination strategy. The Linda virus DI (Fwd_LN^pro^_5′ and Rev_LN^pro^_5′) and the BVDV DI9 (Fwd_BN^pro^_5′ and Rev_BN^pro^_5′) were amplified alongside with a mCherry insert (Fwd_mCherry_5′_LN^pro^_5′ and Rev_mCherry_3′_LN^pro^_5′ as well as Fwd_mCherry_5′_BN^pro^_5′ and Rev_mCherry_3′_BN^pro^_5′) providing suitable overhangs for recombination. The resulting subgenomes were termed Linda-DI-mCherry and BVDV-DI9-mCherry (Fig. 1C). The deletion of the core protein gene in the BAC clone CSFV-Ubi (27) was executed with the aid of oligonucleotides Fwd_SPPcore_3′ and Rev_SPPcore_3′. A second assembly site located within the chloramphenicol acetyltransferase gene (CAT) was used to generate suitable PCR products for homologous recombination. To clearly separate the functions of NS2-3 and mature NS3, a cysteine-to-alanine mutation was inserted in the active center of NS2 of the CSFV-Ubi. This modification was accomplished using oligonucleotides Fwd_NS2_ C_1512_A and Rev_ NS2_ C_1512_A in conjunction with the CAT assembly site primers.

### Transfection, infection, virus harvest, indirect immunofluorescence, and reporter fluorescence assays

Transfecting SK-6 cells with infectious synthetic pestiviral RNAs followed the established protocol, involving genome-length PCRs, SP6 polymerase-mediated transcription of synthetic RNA, and electroporation (32). Protein extracts from transfected cells were prepared 24 hours post-transfection (p.t.) by directly lysing the cells in a loading buffer. Protein extracts from infected cells were prepared 48 hours post-infection (p.i.). To assess viral RNA replication and protein expression post RNA transfection or virus infection, indirect immunofluorescence staining was employed. The procedure involved washing the cells with PBS, fixation with 4% paraformaldehyde, permeabilization with 0.5% Triton-X 100 (Merck, Darmstadt, Germany), and antigen detection via monoclonal antibodies (MAB). E2 expression was assessed via MAB A18 (25). Independent of the expression of the reporter genes, the CSFV and BVDV DIs could be stained with the NS3-specific MAB 8.12.7 (33), kindly provided by E. J. Dubovi, and the Linda virus DI with the MAB 11D5 anti-NS3 (34). Indirect immunofluorescence staining utilized Cy3- or FITC-conjugated goat anti-mouse IgG sera (Dianova; Hamburg, Germany). Cellular nuclei were counterstained with 4′,6-diamidino-2-phenylindole (DAPI; Thermo Fisher Scientific; Waltham, Massachusetts, USA) at a concentration of 1 µg/mL for 5 minutes at room temperature. The mCherry-protein fluorescence signal was directly monitored without immunostaining. Signals were evaluated using a sensitive fluorescence microscope (IX70; Olympus; Tokyo, Japan) and photomicrographs were captured with a monochromatic camera (DFC3000G; Leica; Wetzlar, Germany). All images presented in a single figure were acquired from the same experiment, following identical staining procedures, and using identical microscope settings. Live-cell imaging was performed using an EVOS M5000 system (Thermo Fisher) equipped with a Texas Red LED light cube. Cells were seeded in a cell culture chamber on a glass slide (94.6170.402; Sarstedt; Nümbrecht, Germany), infected with 100 µL supernatant of the transfected cells, and checked at 12 h p.i. for fluorescence signals. Bright-field and red fluorescence images were taken every 15 minutes until the cells were overgrown and dead. The resulting images were overlayed and compiled into time-lapse movies (three frames per second).

### SDS-PAGE and immunoblotting

Cell lysates underwent protein separation in sodium dodecyl sulfate-polyacrylamide tricin gel electrophoresis (SDS-PAGE) (35). Following electrophoresis, proteins were transferred to either nitrocellulose (Pall Corporation; Port Washington, NY) or polyvinylidene fluoride membranes (Immobilon-FL; Sigma). Membranes were then blocked using 4% dried skim milk (wt/vol; Carl Roth GmbH; Karlsruhe, Germany) in PBS containing 0.1% Tween 20 (vol/vol; Carl Roth GmbH). NS3 expression was detected using MAB 8.12.7 (33), generously provided by E. J. Dubovi (Cornell University, Ithaca, NY), whereas E2 was detected using MAB A18. Subsequently, the membranes were incubated with MABs as specified, followed by the application of HRP-conjugated goat anti-mouse IgG (Dianova) and chemiluminescence reagent (ECL-Plus; Perkin Elmer; Waltham, MA) for signal detection. Photon emission was captured using an imaging system (ChemiDoc; Bio-Rad, Hercules, CA, USA).

### Quantification of infectious doses

The 50% tissue culture infectious dose (TCID_50_) of sterile filtered (0.45 µm, Minisart RC4; Satorius; Göttingen, Germany) supernatants from tissue cultures was determined in triplicate via an end-point dilution assay (EPDA) using SK-6 cell monolayers and 96-well plates. Following a 4-day incubation, the cells were fixed and subjected to immunostaining as detailed above. After infection with DIs, only individual infected cells were detectable, as these genomes could not package RNA independently without the aid of a helper virus. Since DIs were serologically indistinguishable from the packaging helper viruses, the infectious doses could not be determined without a special technique. For the quantification of DIs, we added wtCSFV 6 hours post-infection with DIs to these cultures using a multiplicity of infection (MOI) of 1. This secondary infection facilitated DI spread, streamlining titration assessment through plaque counting. Nevertheless, the evaluation remained rather difficult; hence, we introduced a reporter gene that ensured objective quantification via fluorescence. Plaque counting was considerably simplified using the reporter DIs, as the infected cells were stained by mCherry expression and thus stood out strongly as a red cluster of dead cells from the monolayer. Virus titers were calculated utilizing the Spearman-Kaerber algorithm. Additionally, SK-6 cells were infected with 100 µL of the respective supernatants without the use of additional helper viruses to visualize the packaging efficiencies of the experiments. Hence, only individual infected cells became visible, where successful DI packaging occurred in the absence of helper virus RNA packaging.

### *Trans*-packaging assays

For the *trans*-packaging experiments, 0.5 µg of synthetic RNAs from subgenomes and helper viruses were either transfected alone or in combination via electroporation into 1 × 10^6^ naïve SK-6 cells. The RNA was mixed with 300 µL of cell suspension and then pulsed once in a 0.2 cm cuvette at 0.18 kV and 950 µF (GenPulser II, Bio-Rad). An aliquot of 10% from the transfected cells was seeded onto a 24-well plate and incubated for 24 hours. These cells were fixed and subjected to indirect immunofluorescence assays to assess and document the transfection efficiency. The remaining cells were seeded into a well of a 6-well plate and incubated for 48 hours. After incubation, the supernatant containing progeny viruses was collected, whereas the cellular monolayer was lysed in SDS-PAGE loading buffer for subsequent western blot analyses. The cell culture supernatants were clarified by centrifugation and filtered through a 0.45 µm syringe attachment device to eliminate any contamination with transfected cells. All experiments were conducted at least three times (biological replicates) and analyzed together with the control transfections as indicated.

## RESULTS

### CSFV helper viruses efficiently *trans*-package replication-competent subgenomic RNAs (DIs), regardless of the presence of the N_2177_Y mutation

We generated a mCherry-tagged version of pestiviral DIs (CSFV-DI-mCherry, Linda-DI-mCherry, and BVDV-DI9-mCherry), mirroring the genome organization of DI9 of BVDV-1 (31), but with mCherry’s coding sequence inserted after the 15th amino acid of N^pro^. This modification allows for clear differentiation of this subgenome from the CSFV helper viruses. Previous studies by other researchers had introduced similar N^pro^-reporter pestiviruses (36, 37). For our initial *trans*-packaging experiments, we utilized the wild-type ncp CSFV (wtCSFV), the reporter DI without additional mutations (CSFV-DI-mCherry), and the reporter DI harboring the compensatory mutation N_2177_Y in NS3 (CSFV-DI-N_2177_Y-mCherry), which can compensate for the lack of core protein. Control experiments were conducted concurrently using unlabeled CSFV-DI and CSFV-DI-N_2177_Y to rule out any adverse effects of the mCherry reporter gene. The evaluation of the *trans*-packaging of these cytopathogenic DIs without reporter genes was performed by counting the DI-plaques after superinfection with ncp CSFV (see M&M). Differentiating immunofluorescence staining could not be performed as there were no antigenic differences between the helper virus and the DI. Since there were no greater disparities observed between the titer of reporter gene-free DIs and the mCherry labeled subgenomes, only the reporter DIs were utilized in the subsequent experiments presented here (Table 3).

**TABLE 3:** Packaging and *trans*-packaging activity after transfection of wtCSFV and CSFV-Dis

Helper viruses and DIs	Progeny virus production (Arithmetic mean)
wtCSFV	2.2 × 10^6^ TCID_50_/mL
CSFV-DI	n.d.^*a*^
CSFV-DI-mCherry	n.d.^*a*^
CSFV-DI-N_2177_Y	n.d.^*a*^
CSFV-DI-N_2177_Y-mCherry	n.d.^*a*^
wtCSFV + CSFV-DI	3.2 × 10^5^ TCID_50_/mL
	1.3 × 10^4^ TCID_50_/mL
wtCSFV + CSFV-DI-mCherry	8.9 × 10^5^ TCID_50_/mL
	1.7 × 10^4^ TCID_50_/mL
wtCSFV + CSFV-DI-N_2177_Y	6.0 × 10^5^ TCID_50_/mL
	8.3 × 10^3^ TCID_50_/mL
wtCSFV + CSFV-DI-N_2177_Y-mCherry	4.7 × 10^5^ TCID_50_/mL
	1.3 × 10^4^ TCID_50_/mL

^*a*^ n.d. means not detectable.

**Fig 2:**
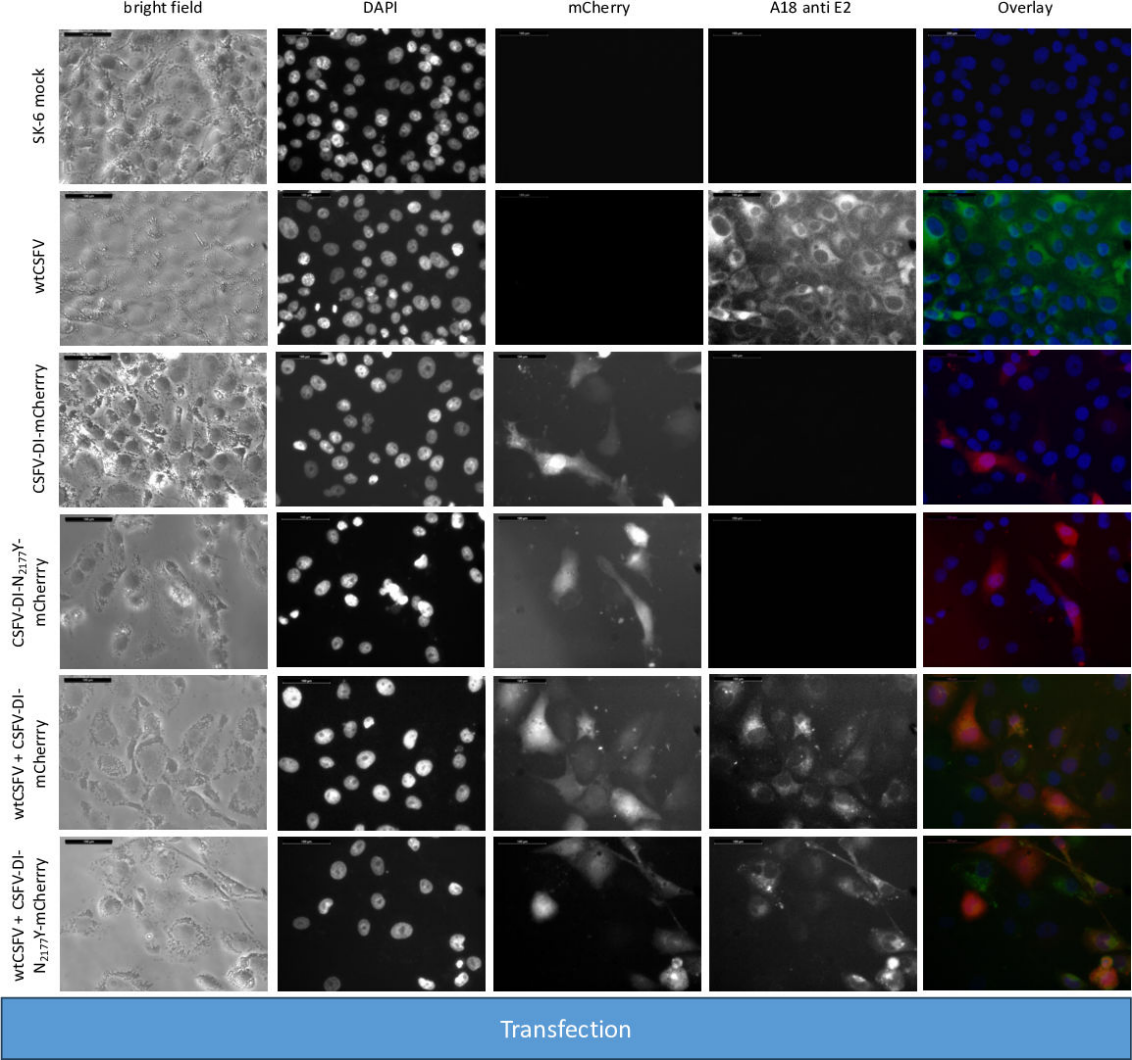
Transfection of SK-6 cells with wtCSFV and DIs. Synthetic RNA of wtCSFV and the reporter DIs CSFV-DI-mCherry-N_2177_Y (with a compensatory NS3 mutation) and CSFV-DI-mCherry (without the mutation) were transfected via electroporation. After 24 hours, the cells were fixed and immunostained for the E2 protein. Nuclei were counterstained with DAPI, and fluorescence was captured using a monochromatic camera at 20-fold magnification. To enhance visualization, images were overlaid with DAPI in blue, mCherry in red, and E2 in green. Notably, double-transfected cells exhibited yellow staining. Naïve SK-6 cells served as a control for the E2 staining. A size marker of 100 µm length was included in the upper left corner for reference.

**Fig 3:**
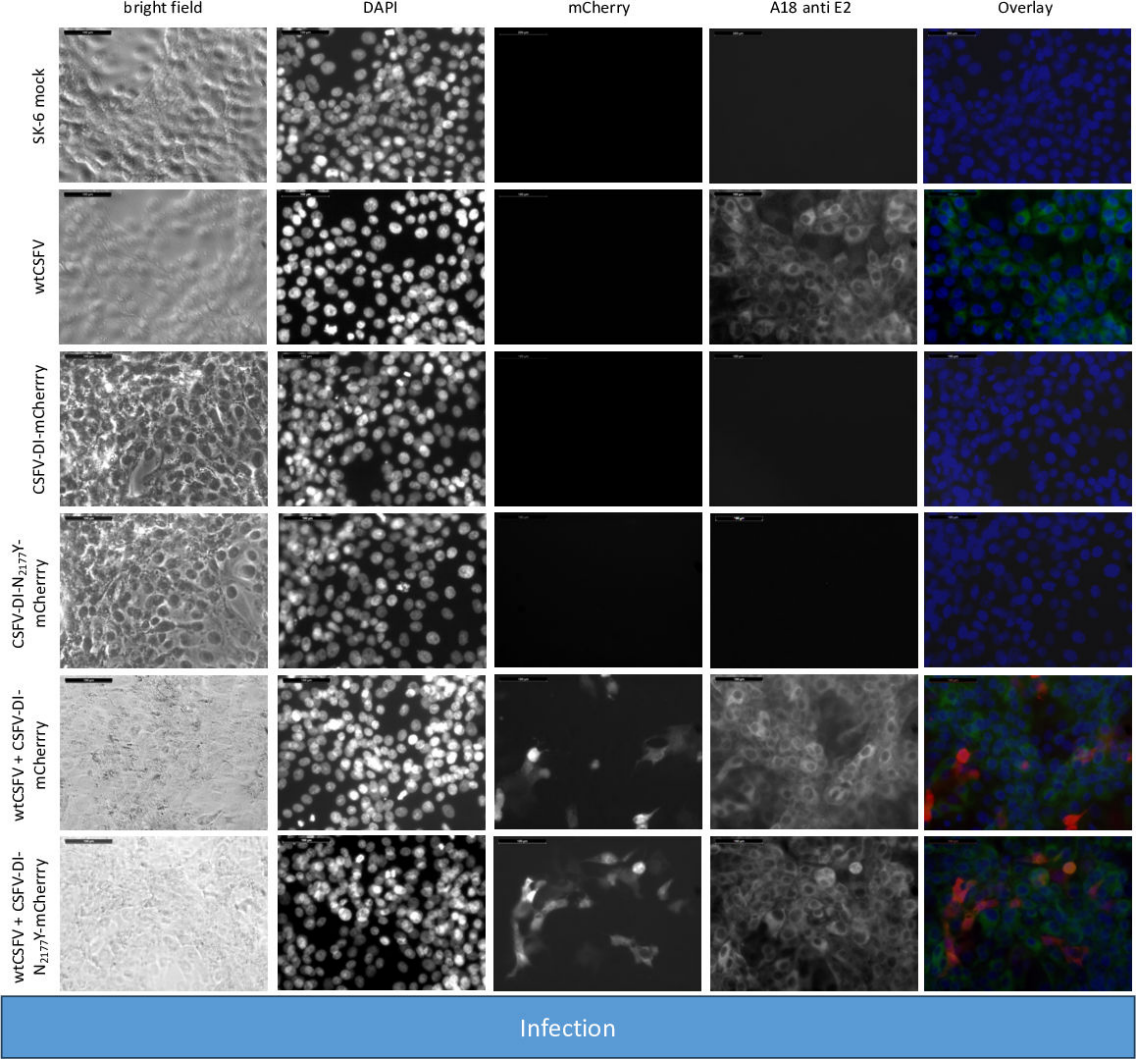
Infecting SK-6 cells with cell culture supernatants from wtCSFV and DI transfected cells. Naïve SK-6 cells were inoculated with 100 µL of sterile filtered cell culture supernatant harvested 48 hours post-transfection. After a 48-hour incubation period, the cells were fixed and immunostained for the E2 protein, with nuclei counterstained using DAPI. Fluorescence was captured via a monochromatic camera at 20-fold magnification. For enhanced visualization, images were overlaid, presenting DAPI in blue, mCherry in red, and E2 in green. Noteworthy is the discernible focus formation of DIs, evident after co-packaging with the helper virus. A size marker, 100 µm in length, was incorporated in the upper left corner for reference.

For the experiment, RNA of the helper virus (wtCSFV) and the DIs were individually or jointly transfected into SK-6 cells. Staining of the cells transfected with wtCSFV 24 hours post-transfection, using an E2-specific antibody, indicated E2-expression in all cells, suggesting efficient transfection (Fig. 2). Infectious progeny virus production of wtCSFV was evaluated 48 hours post-transfection by infecting naïve cells (Fig. 3) and quantified using EPDA. The mean infectious titer of wtCSFV was determined to be 2.2 × 10^6^ TCID_50_/mL (Table 3). Transfecting the mCherry-CSFV-DI alone (with or without the NS3 mutation N_2177_Y) led to robust transfection efficiencies, evident from the intense fluorescence of the mCherry reporter in many cells (Fig. 2). However, no progeny viruses could be produced by the DIs, as the subgenomes lack the structural protein genes (Fig. 3).

**Fig 4:**
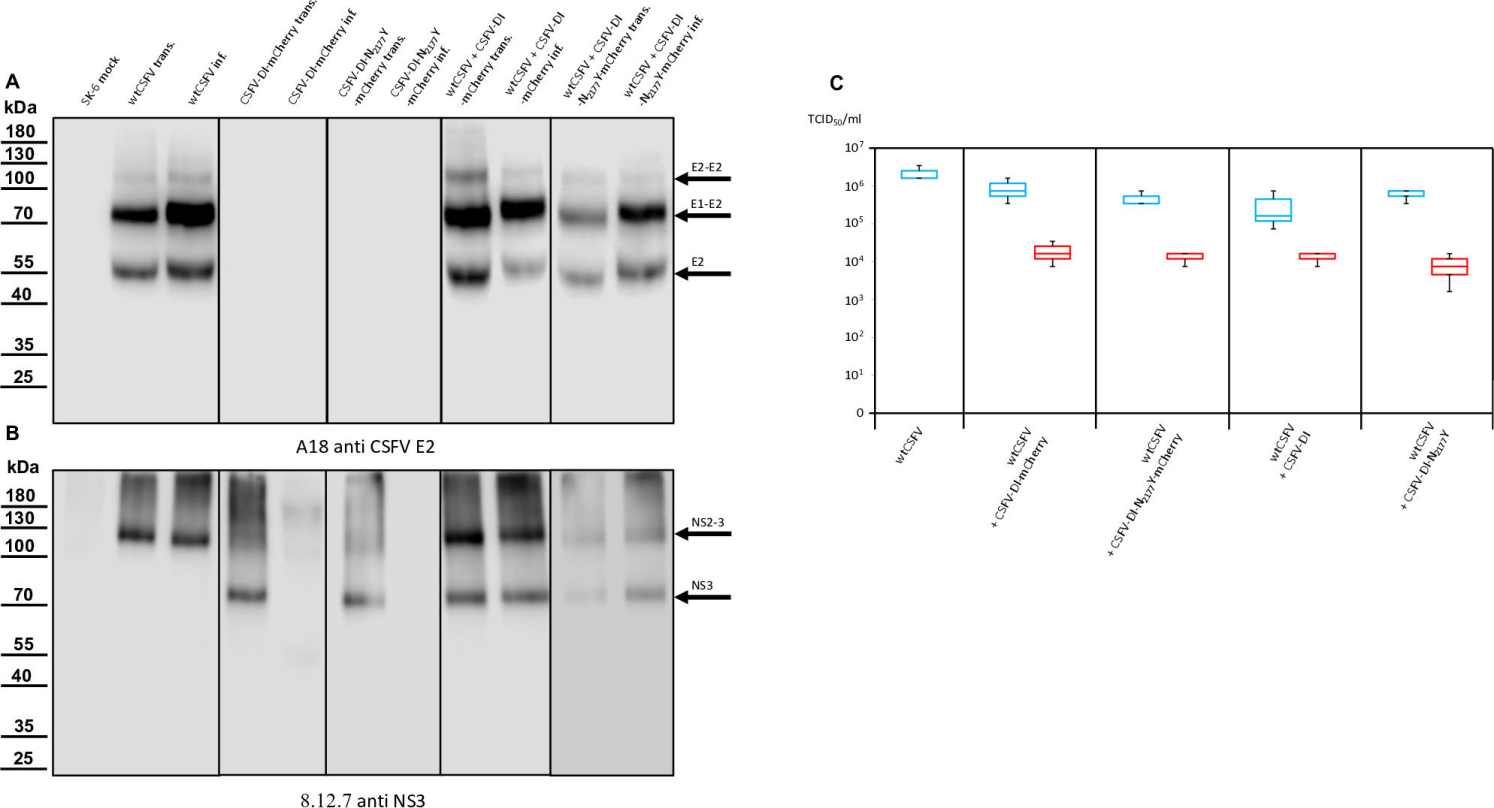
Western blot analyses conducted on cells transfected or infected with wtCSFV and DIs. (**A**) E2 expression was examined using A18 anti-CSFV E2. No signal was detected in naïve SK-6 cells or cells solely transfected with DIs or infected with cell culture supernatant from DI transfection. However, transfection of wtCSFV RNA and infection with the supernatant produced characteristic bands of E2 at 55 kDa (monomer), 80 kDa (heterodimer), and 120 kDa (homodimer). The same band pattern was observed after co-transfection with the DIs and infection. (**B**) NS3 expression was analyzed using 8.12.7 anti-NS3. Transfection of wtCSFV and infection with the supernatant resulted in typical NS2-3 bands at 125 kDa. The NS2-3 band was also present after co-transfection and infection with the DIs. Transfection of DI-RNA alone yielded a band at 80 kDa (mature NS3), which was also observed after *trans*-packaging with the helper virus but was absent when naïve cells were inoculated with supernatant from the DIs alone. (**C**) Box and whisker plots depicting progeny virus production and *trans*-packaging. The 50% tissue culture infection dose (TCID_50_) was determined for both the helper virus (blue box) and the *trans*-packaged DIs (red box). Forty-eight hours post-transfection, the supernatants were harvested and used to infect naïve cells in dilution series, followed by immunostaining against the E2 protein to quantify the helper virus. The fluorescence of the mCherry reporter was used to quantify DI production in cultures superinfected with wtCSFV to allow DI-plaque formation. Titers were calculated using the Spaerman-Kärber method based on the results of three independent RNA transfections, each analyzed in three dilution series.

The transfection efficiency of both wtCSFV and the subgenomic DIs (CSFV-DI-mCherry and CSFV-DI-N_2177_Y-mCherry) appeared comparable 24 hours post-transfection when electroporated together as the majority of cells showed E2 and reporter gene expression at the same time (Fig. 2). Subsequent infection of naïve cells with the supernatant collected 48 hours post-transfection revealed efficient production of progeny virus, as evidenced by immunofluorescence staining using the E2-specific antibody (Fig. 3). Concurrently, a robust *trans*-packaging yield of the reporter DIs was evidenced by the fluorescence of the mCherry reporter (Fig. 3). The average titer of wtCSFV, determined through titration and E2-specific staining of infected cells, exhibited an approximate 60%–95% reduction, reaching 8.9 and 4.7 × 10^5^ TCID_50_/mL, respectively (Fig. 4C). The decline might be attributed to the pronounced cytopathogenicity of the DIs, leading to significant cell death within the 48 hours of incubation. Substantial yields of *trans*-packaged DIs were achieved, with titers exceeding 1.0 × 10^4^ TCID_50_/mL for CSFV-DI-mCherry and CSFV-DI-N_2177_Y-mCherry. Although a similar titer difference was observed between the reporter gene-free DIs, no statistically significant difference was discerned for the N_2177_Y mutation, which can be attributed to the variation within the biological replicates of the experiments.

Live cell imaging showed different propagation kinetics when the supernatants of wtCSFV/CSFV-DI-mCherry or wtCSFV/CSFV-DI-N_2177_Y-mCherry transfected cells were added to naïve SK-6 cells (Movies S1 and S2). In the wtCSFV/CSFV-DI-mCherry combination, the primary infected cells started to spread the infection to neighboring cells after approximately 24 hours. Additional infected cells appeared throughout the monolayer, with further spread observed after 35 hours and long-distance spread after approximately 60 hours. After about 72 hours, the entire monolayer showed signs of disruption and almost all cells were infected with the cytopathogenic pathogen. In contrast, CSFV-DI-N_2177_Y-mCherry showed a significantly different dynamic, when *trans*-packaged by the wild-type virus. Although strong signals from a detached doublet were already visible 12 hours after infection and a first contact infection was already observed after 24 hours, the subsequent infection process was delayed. Clear signals in neighboring cells and plaque formation were only visible after about 57 hours. From 80 hours p.i., progeny plaques started to form, but the infection process came to a halt due to the limited growth conditions in the microscope chamber, resulting in cell death and destruction of the monolayer. Despite these obvious differences in infection kinetics after infection and *trans*-complementation, minor differences were seen in *trans*-packaging after RNA transfection.

Previous investigations have indicated that the N_2177_Y mutation also facilitates the packaging of CSFV genomes in the presence of the core. However, despite the presence of core protein in the cell, the mutation completely prevents the incorporation of core into virions (25). Hence, the *trans*-packaging of the CSFV-DI-N_2177_Y-mCherry in the presence of core protein was not unexpected. Western blot analyses of transfected and infected cells yielded results consistent with immunofluorescence staining. The E2-specific antibody successfully stained the helper virus, distinguishing it from the DIs, which lack E2 expression (Fig. 4A).

Negative control and DI-transfected cells showed no E2 expression, whereas those transfected with wtCSFV displayed distinct E2 band patterns. E2, existing in monomeric, homodimeric (E2-E2), and heterodimeric (E1-E2) forms exhibited bands at 48 kDa (monomer), 80 kDa (heterodimer), and 120 kDa (homodimer). Naïve cells exposed to supernatants derived from wtCSFV-transfected cells also exhibited robust E2 expression. DI-infected cells were discernible by the strong expression of mature NS3 (80 kDa), whereas wtCSFV-infected cells displayed a very weak NS3 band and a strong 125 kDa NS2-3 band (Fig. 4B). Co-transfection of wtCSFV and DIs yielded the 125 kDa band together with a prominent 80 kDa signal. Sole DI transfections lacked infectivity in supernatants, whereas helper virus co-transfection-derived supernatants exhibited the typical 125 and 80 kDa pattern.

### A coreless CSFV carrying mutation N_2177_Y is unable to *trans*-package DIs bearing wild-type NS3 sequences

**Fig 5:**
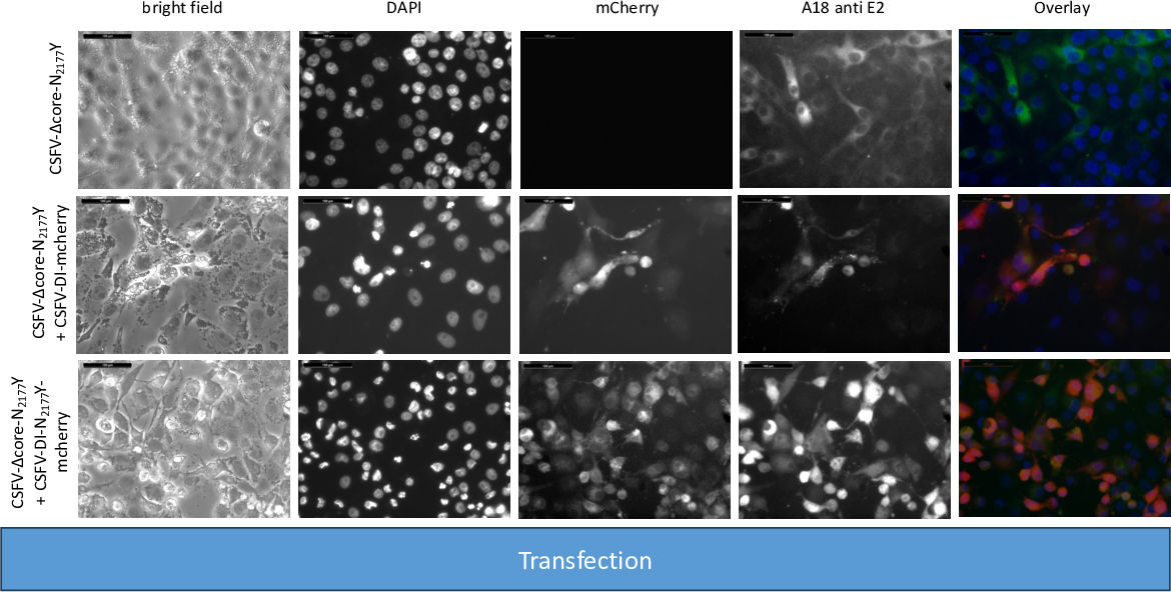
Transfection of SK-6 cells with a coreless CSFV bearing compensatory mutation N_2177_Y (CSFV-Δcore-N_2177_Y) and DIs. Synthetic RNA of CSFV-Δcore-N_2177_Y and the reporter DIs CSFV-DI-mCherry-N_2177_Y (also containing the compensatory NS3 mutation) and CSFV-DI-mCherry (bearing wild-type NS3 sequences) were transfected via electroporation. Following a 24 hour incubation period, the cells were fixed and subjected to immunostaining for the E2 protein. Nuclei were counterstained with DAPI, and fluorescence signals were captured using a monochromatic camera at 20-fold magnification. For visualization, the images were superimposed with DAPI in blue, mCherry in red, and E2 in green. Notably, a greater number of cells exhibited mCherry fluorescence in the case of CSFV-DI-mCherry-N_2177_Y. A size marker of 100 µm length was included in the upper left corner for scale reference.

**Fig 6:**
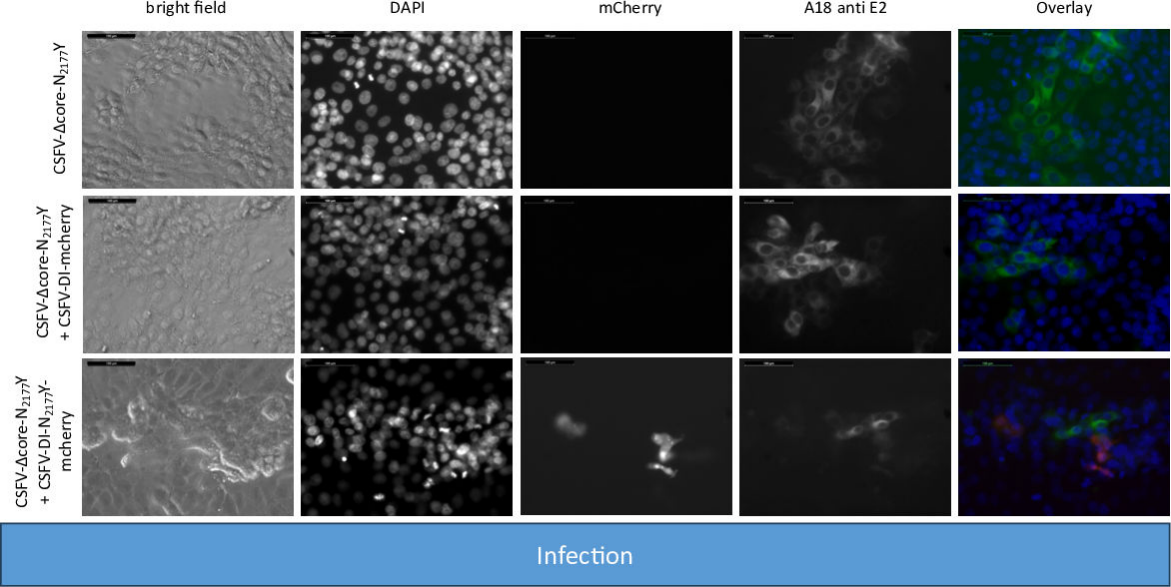
Inoculating naïve SK-6 cells with cell culture supernatants from transfections of coreless CSFV bearing compensatory mutation N_2177_Y (CSFV-Δcore-N_2177_Y) and from DI co-transfections. After a 48 hour post-transfection incubation, 100 µL of sterile filtered cell culture supernatant was used for inoculation of naïve cells. After another 48 hour incubation, the cells were fixed and immunostained for the E2 protein, whereas nuclei were counterstained using DAPI. Captured at 20-fold magnification with a 100 µm size marker, the images were overlaid, showing DAPI in blue, mCherry in red, and E2 in green. Notably, only CSFV-DI-N_2177_Y-mCherry was *trans*-packaged by the CSFV-Δcore-N_2177_Y helper virus.

To investigate how the NS3 N_2177_Y mutation influences *trans*-packaging in the absence of the core protein, RNAs from the two DIs were transfected into SK-6 cells alongside the coreless CSFV RNA carrying mutation N_2177_Y (CSFV-Δcore-N_2177_Y). As a control, transfection of CSFV-Δcore-N_2177_Y alone was performed. Immunofluorescence staining with an E2-specific antibody revealed the replication of CSFV-Δcore-N_2177_Y in the majority of cells 24 hours post-transfection, indicating efficient transfection, genome replication, and protein expression (Fig. 5). Infectious progeny virus production was observed upon infecting naïve cells with the supernatant of CSFV-Δcore-N_2177_Y transfected cells (Fig. 6). The mean infectious titer of CSFV-Δcore-N_2177_Y 48 hours post-transfection was calculated at 1.3 × 10^5^ TCID_50_/mL (Table 4). A reduction in titer compared with wtCSFV was noted earlier, indicating that coreless packaging by mutation N_2177_Y is less efficient than packaging involving wild-type NS3 and core (25). A high transfection efficiency of CSFV-Δcore-N_2177_Y RNA transfected together with subgenomic DI RNAs (with or without mutation N_2177_Y) was observed after electroporation (Fig. 5). Progeny virus production was evident in immunofluorescence staining using the E2-specific antibody after co-transfection. Due to the lower titer and resulting slower growth of CSFV-Δcore-N_2177_Y, the focus size was also reduced. CSFV-Δcore-N_2177_Y reached average titers of 2.8 and 6.0 × 10^4^ TCID_50_/mL, respectively, after co-transfections. The further decrease in titer might again be attributable to the pronounced cytopathogenicity of the DIs. No *trans*-packaging of the CSFV-DI-mCherry was observed when examining the fluorescence of the mCherry reporter (Fig. 6). In contrast, the presence of the N_2177_Y mutation facilitated packaging of CSFV-DI-N_2177_Y-mCherry, reaching average titers of 9 × 10^3^ TCID_50_/mL. These findings indicate that the NS2-3 of the helper virus containing mutation N_2177_Y enables self-packaging in the absence of the core. However, this virus is not active in *trans*-packaging a subgenome encoding wild-type NS3. In contrast, the subgenome containing mutation N_2177_Y in the mature NS3 was efficiently *trans*-packaged by the coreless helper virus, which itself encodes mutation N_2177_Y.

**TABLE 4:** Packaging and *trans*-packaging activity after transfection of CSFV-Δcore-N_2177_Y and CSFV-Dis

Helper viruses and DIs	Progeny virus production
CSFV-Δcore-N_2177_Y	1.3 × 10^5^ TCID_50_/mL
CSFV-Δcore-N_2177_Y CSFV-DI-mCherry	2.8 × 10^4^ TCID_50_/mL
	n.d.^*a*^
CSFV-Δcore-N_2177_Y CSFV-DI-N_2177_Y-mCherry	6.0 × 10^4^ TCID_50_/mL
	9.0 × 10^3^ TCID_50_/mL

^*a*^ n.d. means not detected.

Live cell imaging showed a marked delay in plaque formation and spreading of CSFV-DI-N_2177_Y-mCherry when packaged by CSFV-Δcore-N_2177_Y (Movie S3). After initial infection, weak mCherry signals appeared in a single cell, which subsequently detached from the plate surface and was lifted out of the monolayer. This was followed by a prolonged gap phase in which no neighboring cells were infected, suggesting that CSFV-Δcore-N_2177_Y established infection more slowly before DI spread became possible. Only after about 57 hours p.i., stronger signals and plaque formation appeared in neighboring cells. Although further spread, both near and far, began at approximately 80 hours p.i., the overall plaque formation of the DI was limited in this combination as the cells were already overgrown and the monolayer perished.

**Fig 7:**
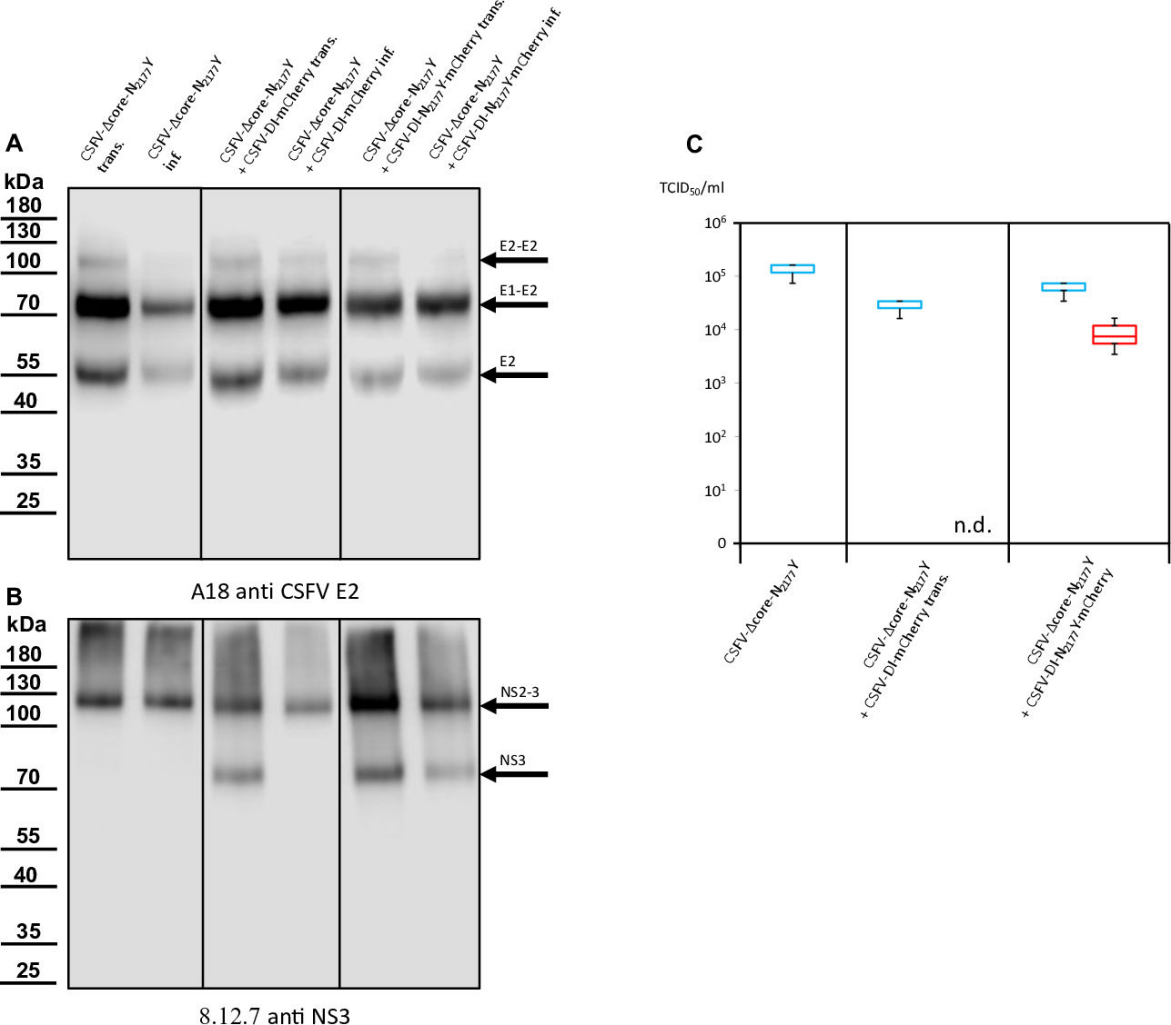
Western blot analyses performed on cells transfected or infected with CSFV-Δcore-N_2177_Y and DIs. (**A**) Expression of CSFV E2, marked by bands at 55, 80, and 120 kDa, was consistently observed post-transfection and infection of/with CSFV-Δcore-N_2177_Y, regardless of DIs co-transfection. (**B**) NS2-3 expression, with a distinctive band at 125 kDa, was evident after transfection and infection of/with CSFV-Δcore-N_2177_Y, irrespective of DIs co-transfection. The mature NS3, represented by an 80 kDa band, emerged following DIs co-transfection. However, this band only appeared upon infection of naïve cells in the case of CSFV-DI-N_2177_Y-mCherry, indicating successful *trans*-packaging by the CSFV-Δcore-N_2177_Y helper virus. (**C**) Box and whisker plots depicting progeny virus production and *trans*-packaging in the absence of core protein. The 50% tissue culture infection dose (TCID_50_) was determined for both the helper virus (blue box) and the *trans*-packaged DI (red box). Supernatants were applied to naïve cells in a dilution series 48 hours post-transfection, followed by immunostaining against the E2 protein to quantify the helper virus. A measurement of mCherry fluorescence after superinfection of the cultures with wtCSFV was used to quantify DI production. Titers were calculated using the Spaerman-Kärber method based on the results of three independent RNA transfections, each analyzed in three dilution series. The abbreviation n.d. means not detected.

Concurrent western blot analyses also indicate the production of infectious virus by CSFV-Δcore-N_2177_Y (Fig. 7A). The sample of the infected cells exhibited a reduced E2 expression compared with cells transfected with synthetic RNA of CSFV-Δcore-N_2177_Y, indicating reduced infectivity and spread of the coreless virus. This was also demonstrated in the NS2-3 bands of the same sample, albeit less prominently discernible (Fig. 7B). The CSFV-Δcore-N_2177_Y also depicted characteristic E2 bands in the western blots of transfections and infections following co-transfection with the DIs. The DI with wild-type NS3 (CSFV-DI-mCherry) failed to propagate in the absence of core, as already evident from the absence of spread in the transfected cultures. Analysis of the NS3 expression underlined a complete *trans-*packaging fail showing no visible band of mature NS3 after infection of naïve cells with the supernatant. In contrast, the co-transfection using the DI-carrying compensatory NS3 mutation N_2177_Y (CSFV-DI-N_2177_Y-mCherry) exhibited a band of mature NS3. This result raised the question of whether the NS3 mutation N_2177_Y must be present in both NS2-3 and mature NS3 to allow genome packaging in the absence of the core.

### The N_2177_Y mutation in the NS3 of DIs allows coreless *trans*-packaging, whereas a helper virus with wild-type NS2-3 is not packaged

**Fig 8:**
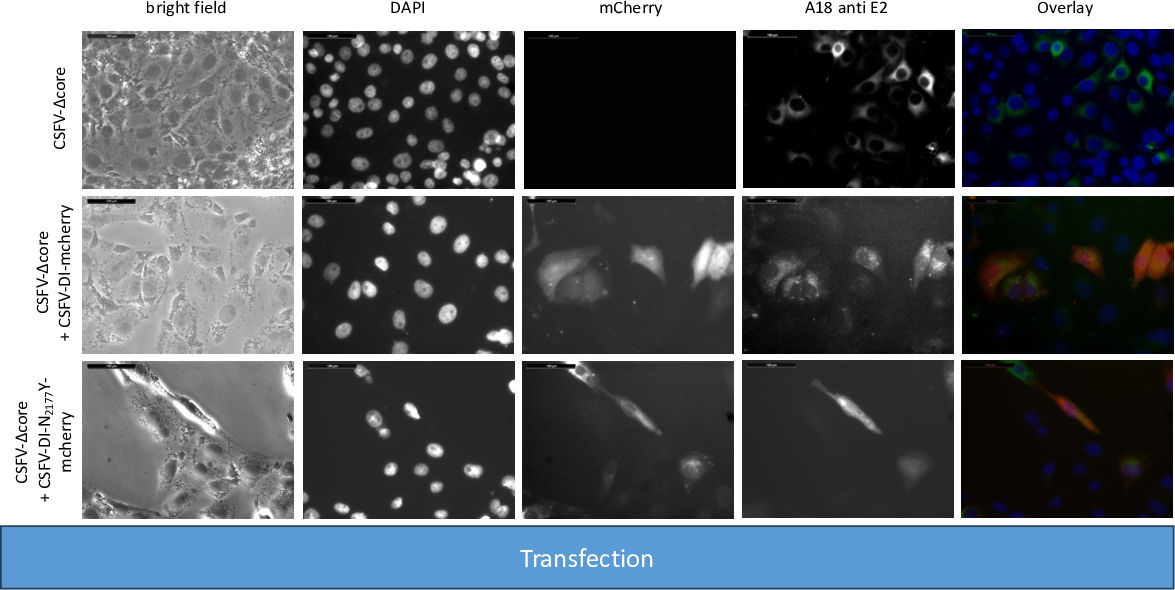
SK-6 cells transfected with RNAs of CSFV-Δcore and DIs. Synthetic RNA of CSFV-Δcore lacking compensatory NS3 mutations and the reporter DIs CSFV-DI-mCherry-N_2177_Y (bearing the compensatory NS3 mutation) and CSFV-DI-mCherry (without NS3 mutation) were co-transfected via electroporation. After 24 hours, the cells were fixed and immunostained for the E2 protein, with nuclei counterstained using DAPI. Fluorescence microscopy was performed at 20-fold magnification, with overlay images displaying DAPI in blue, mCherry in red, and E2 in green, alongside a size marker of 100 µm.

To probe the significance of N_2177_Y in the NS2-3 precursor, we conducted an experiment with a coreless helper virus bearing wild-type NS2-3 sequences (CSFV-Δcore). RNAs of this packaging defect genome alone and alongside the DIs were transfected and analyzed as before. Immunofluorescence staining with an E2-specific antibody revealed replication of CSFV-Δcore only in single cells and doublets 24 hours post-transfection, whereas no focus formation was observed, indicating efficient RNA transfection, genome replication, and protein expression (Fig. 8).

**Fig 9:**
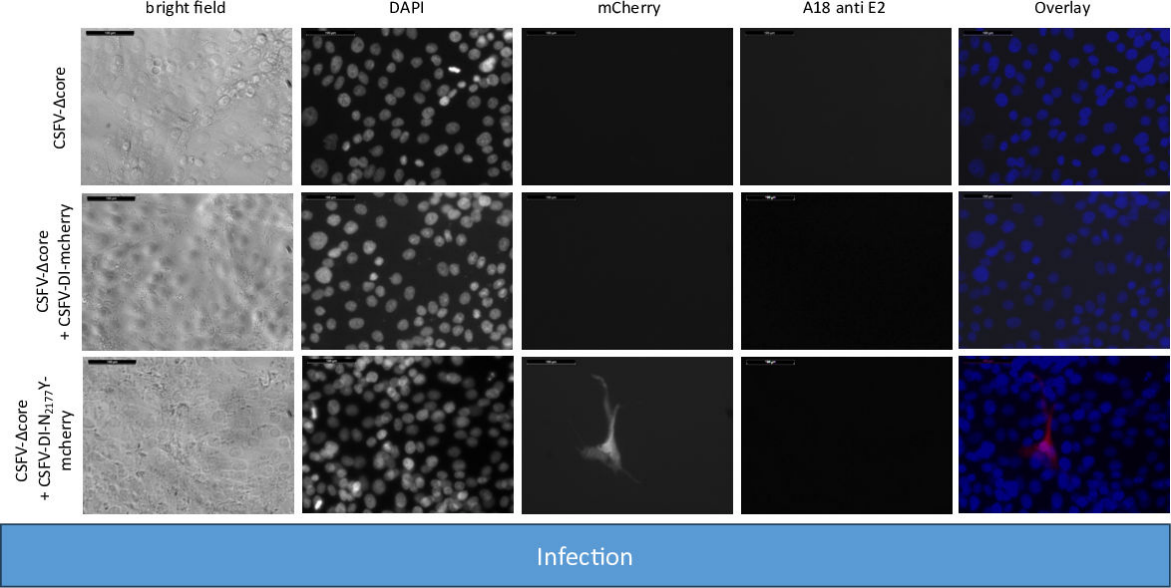
Inoculation of SK-6 cells with cell culture supernatants from CSFV-Δcore and DI transfected cells. SK-6 cells were infected with 100 µL cell culture supernatant, followed by 48 hours of incubation. After fixing, the cells were immunostained for the E2 protein and counterstain using DAPI. The results showed that CSFV-Δcore failed to produce progeny virus, regardless of DI co-transfection. The co-transfection of CSFV-DI-mCherry resulted in no mCherry-fluorescence positive cells. However, individual red cells emerged post-infection with supernatants of CSFV-DI-N_2177_Y-mCherry co-transfections indicating successful *trans*-packaging by the coreless helper virus.

In line with previous findings, no infectious progeny virus was detected upon inoculation of naïve cells with the cell culture supernatant from CSFV-Δcore transfected cells (Fig. 9). Co-transfection of CSFV-Δcore with the DIs and subsequent staining using an E2-specific antibody demonstrated replication of the genome following transfection but detected no infectious progeny virus production following infection indicating that neither the mature wild-type NS3 nor the N_2177_Y mutated NS3 of the DIs could restore the packaging defect of the coreless helper virus.

**Fig 10:**
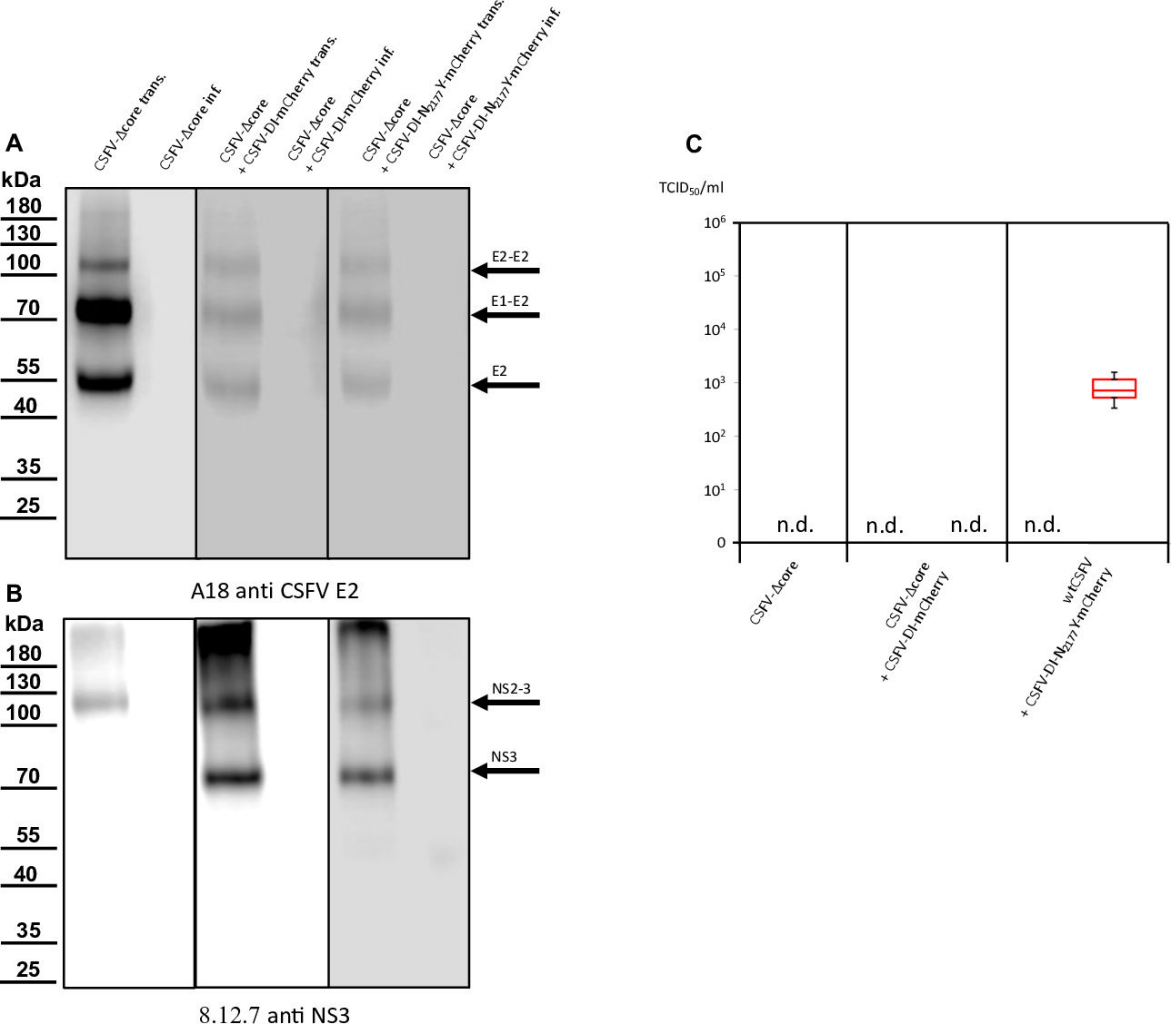
Western blot analyses conducted on cells transfected or infected with CSFV-Δcore and DIs. (**A**) Expression of CSFV E2, evident through bands at 55, 80, and 100 kDa, was consistently observed post-transfection. However, in infection experiments, E2 expression was lacking, indicating no progeny virus production of CSFV-Δcore regardless of DI co-transfection. (**B**) NS2-3 expression, marked by a band at 125 kDa, was evident after transfection of CSFV-Δcore but absent in all infection experiments. Mature NS3, characteristic of DIs, represented by an 80 kDa band, was present following DIs transfection. However, this band was also not visible following infection. Successful *trans*-packaging was documented by the fluorescence of mCherry. The absence of the NS3 band in CSFV-DI-N2177Y infected cells might be attributed to the overall low number of infected cells and the absence of a helper virus, which might have increased the number of DI-infected cells. (**C**) Box and whisker plots depicting progeny virus production and *trans*-packaging in the absence of core protein and compensatory mutation in the NS2-3 of the helper virus. Packaging of helper viruses was not detected (n.d.). The 50% tissue culture infection dose (TCID50) was determined for the *trans*-complemented DI (red box). A measurement of mCherry fluorescence after superinfection of the cultures with wtCSFV was used to quantify DI production. Titers were calculated using the Spaerman-Kärber method based on the results of three independent RNA transfections, each analyzed in three dilution series.

The fluorescence of mCherry was visible after transfection of both DIs. As anticipated from the previous experiments, no packaging of the CSFV-DI-mCherry with the wild-type NS3 was detected after the infection of naïve cells. In contrast, the NS3 mutation in the CSFV-DI-N_2177_Y-mCherry still facilitated packaging of the DI, when co-electroporated with CSFV-Δcore without the N_2177_Y mutation in NS2-3. However, the CSFV-DI-N_2177_Y-mCherry reached a very low average titer of 8.9 × 10^2^ TCID50/mL post co-transfection (Table 5). The relatively lower titer of the DI bearing the compensatory mutation might be caused by the packaging defect of the helper virus (CSFV-Δcore), which exhibited no spread in the transfected cultures, thus reducing the number of cells with replication of DI and the helper virus. Western blot analyses revealed strong E2 expression from the non-cytopathogenic CSFV-Δcore when transfected alone (Fig. 10A). However, due to the absence of infectivity in the produced supernatant, no E2 expression was observed upon inoculation of naïve cells.

**TABLE 5:** Packaging and *trans*-packaging activity after transfection of CSFV-Δcore and CSFV-Dis

Helper viruses and DIs	Progeny virus production
CSFV-Δcore	n.d.^*a*^
CSFV-Δcore CSFV-DI-mCherry	n.d.^*a*^
	n.d.^*a*^
CSFV-Δcore CSFV-DI-N_2177_Y-mCherry	n.d.^*a*^
	8.9 × 10^2^ TCID_50_/mL

^*a*^ n.d. means not detected.

Co-transfections with the DIs led to diminished E2 signaling, likely attributed to the absence of viral spread, and DI replication in the transfected cells suppressing CSFV-Δcore replication. No infection of CSFV-Δcore was detectable after co-transfections, resulting in the absence of an E2 signal after inoculation of naive cells. Transfection of CSFV-Δcore revealed a distinct NS2-3 band, whereas co-transfection with the DI exhibited an additional band corresponding to mature NS3. Following inoculation of naïve cells with the supernatants of the transfections, NS3 expression remained undetectable in all cases. We assumed that the 8.9 × 10^2^ infected cells were not sufficient to yield a recognizable signal in the western blot analysis in the case of CSFV-DI-N_2177_Y-mCherry and a packaging-defect helper virus, hindering further spread and focus formation. In summary, mutation N_2177_Y exclusively manifested its compensatory activity in *cis* and proved effective for packaging in the absence of core when provided in NS2-3 or in the mature NS3.

### The N_2177_Y mutation solely compensates for the lack of core proteins when present in the mature NS3

NS2 autoprotease and JIV-cofactor mediated cleavage of the NS2-3 precursor and maturation of NS3 is indispensable for pestiviral replication. Since the helper virus must retain replication competence for efficient *trans*-packaging, both NS3-containing moieties are necessary in such experiments. The use of *trans*-packaging and DIs ensures a clear separation of the functions of mature NS3 from NS2-3 precursor function but represents a somewhat artificial system segregating the structural protein cassette from the genome replication machinery. Since the structural and non-structural components of the packaging machinery are presented on two separate cistrons, it does not faithfully mimic the natural processes of single-stranded RNA viruses, potentially leading to improper assumptions.

Consequently, we opted to validate our findings by utilizing a single viral genome wherein a duplication of the NS3 coding sequences provides functional segregation of an uncleavable NS2-3 precursor and the mature NS3. Even in nature, recombination events sometimes lead to considerable duplications of the non-structural protein genes in pestiviruses. Such alterations typically emerge in cattle persistently infected with BVDV, inducing a lethal condition known as mucosal disease. Incidental recombination events may integrate genes encoding autocatalytic active products, like the viral N^pro^, or host cell-processed proteins, such as ubiquitin. If this occurs at an appropriate site, further RNA recombination and natural selection lead to the formation of viral genomes with a complete replication cassette comprising the genes NS3 to NS5B located downstream of the novel processing signal. Genomes harboring NS3 duplications exhibit boosted replication and cytopathogenicity due to NS2-independent expression of mature NS3 while generating infectious particles autonomously, without requiring a helper virus (38). We utilized a recently presented cytopathogenic CSFV genome (27), wherein a ubiquitin gene was inserted after the first quarter of the NS4B coding sequence, followed by the replication cassette with NS3 to NS5B genes. In this CSFV-Ubi genome, the autocatalytic activity of the NS2 autoprotease was disabled by mutation C_1512_A, ensuring a functional separation of the NS2-3 precursor and the mature NS3 (CSFV-Ubi-C_1512_A).

**Fig 11:**
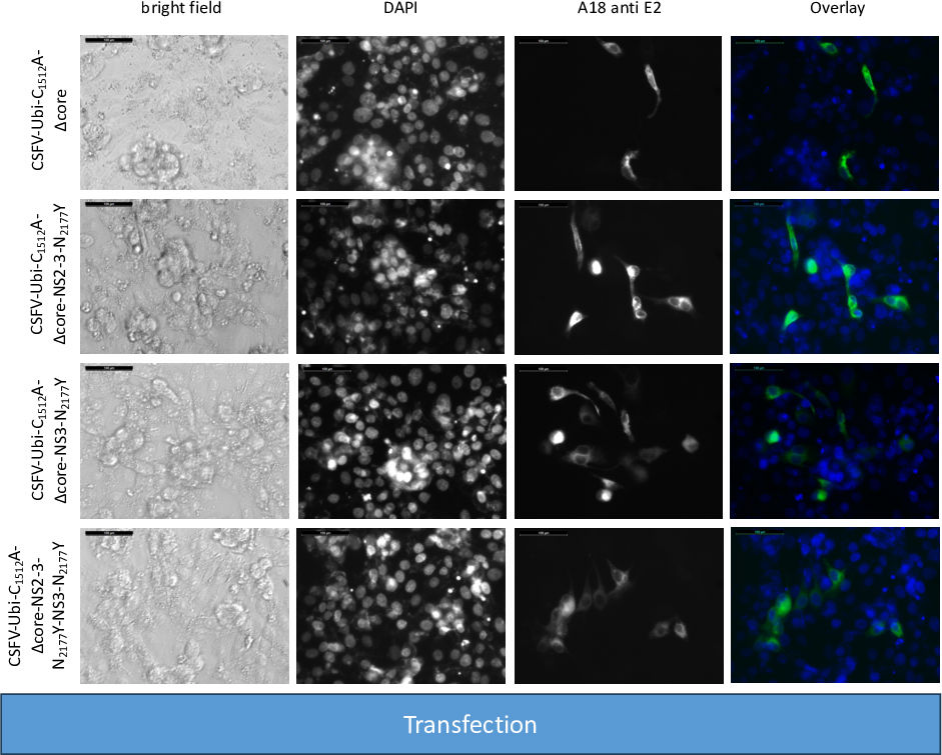
SK-6 cells were transfected with RNAs from coreless CSFVs harboring uncleavable NS2-3 and a genomic duplication of NS3 to NS4B. Synthetic RNAs of the CSFV-Ubi-C_1512_A-Δcore, CSFV-Ubi-C_1512_A-Δcore-NS2-3-N_2177_Y, CSFV-Ubi-C_1512_A-Δcore-NS3-N_2177_Y, and CSFV-Ubi-C1512A-Δcore-NS2-3-N_2177_Y-NS3-N_2177_Y were transfected via electroporation. Following a 24 hour incubation period, the cells were fixed and subjected to immunostaining targeting the E2 protein with DAPI used for nuclei counterstaining. Fluorescence microscopy analysis was conducted at 20-fold magnification accompanied by a 100 µm size marker. An overlay is shown with DAPI displayed in blue and E2 in green. Although CSFV-Ubi-C_1512_A-Δcore and CSFV-Ubi-C_1512_A-Δcore-NS2-3-N_2177_Y exhibited predominantly single transfected cells and doublets, CSFV-Ubi-C_1512_A-Δcore-NS3-N_2177_Y and CSFV-Ubi-C_1512_A-Δcore-NS2-3-N_2177_Y-3-N_2177_Y displayed an increased number of positive cells with formation of small foci.

**Fig 12:**
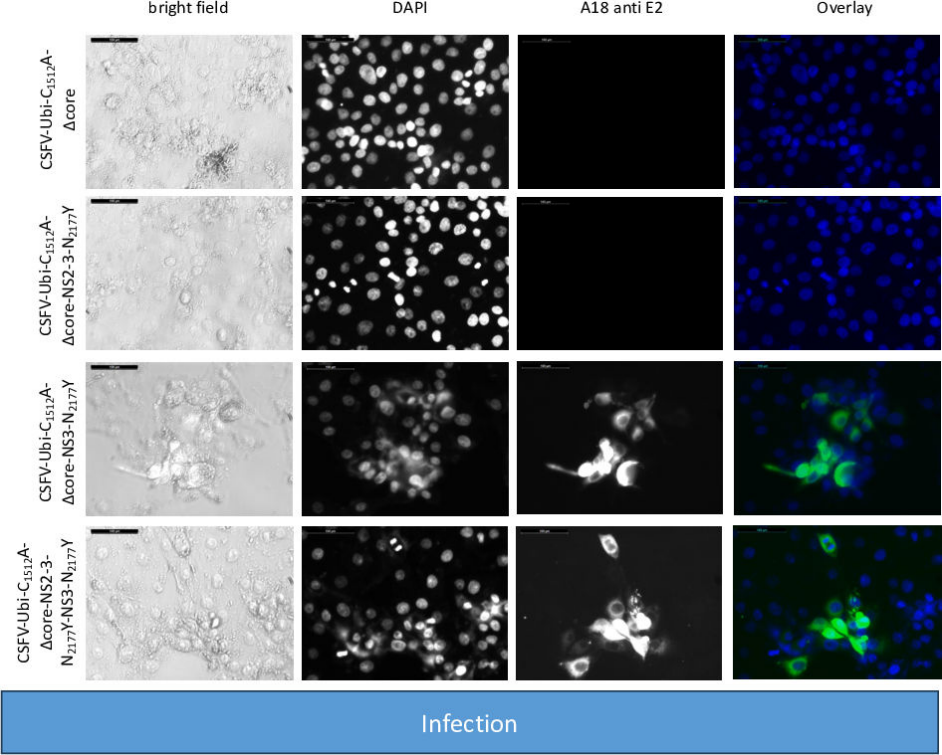
Inoculation of SK-6 cells with cell culture supernatants from coreless CSFVs harboring uncleavable NS2-3 and a genomic duplication of NS3 to NS4B. Following inoculation with 100 µL of cell culture supernatant from CSFV-Ubi-C_1512_A-Δcore, CSFV-Ubi-C_1512_A-Δcore-NS2-3-N_2177_Y, CSFV-Ubi-C_1512_A-Δcore-NS3-N_2177_Y, and CSFV-Ubi-C_1512_A-Δcore-NS2-3-N_2177_Y-NS3-N_2177_Y transfected cells, naïve SK-6 cells were incubated for 48 hours. Subsequently, the cells were fixed and subjected to immunostaining for the expression of E2 protein. The resulting images were supplemented with a DAPI counterstain and included a 100 µm size marker. In the overlay, nuclei are highlighted in blue, whereas E2 immunofluorescence is depicted in green. The absence of green staining indicated the failure of CSFV-Ubi-C_1512_A-Δcore and CSFV-Ubi-C_1512_A-Δcore-NS2-3-N_2177_Y to produce progeny virus. Conversely, CSFV-Ubi-C_1512_A-Δcore-NS3-N_2177_Y and CSFV-Ubi-C_1512_A-Δcore-NS2-3-N_2177_Y-3-N_2177_Y exhibited focus formation and a cytopathogenic phenotype.

Additionally, we deleted the sequence encoding the core protein and introduced the compensating mutation N_2177_Y once in the NS2-3 (CSFV-Ubi-C_1512_A-Δcore-NS2-3-N_2177_Y) and once in the NS3 moiety (CSFV-Ubi-C_1512_A-Δcore-NS3-N_2177_Y). As controls, coreless genomes without compensating mutations (CSFV-Ubi-C_1512_A-Δcore) as well as genomes with both compensating mutations (CSFV-Ubi-C_1512_A-Δcore-NS2-3-N_2177_Y-NS3-N_2177_Y) were analyzed. Immunofluorescence staining unveiled replication of all these viruses documented by an intense E2 staining following RNA transfection (Fig. 11). However, a diminished count of antigen-positive cells is noted across all four transfections compared with prior experiments. This can be attributed to the challenges in DNA preparation and reduced efficacy of genome-length PCR, stemming from the inefficient preparation of BAC and from the complexity of these genomes with duplicated parts compared with pestiviral cDNA coded on plasmids. Despite equating RNA quantities, the lower RNA quality led to lower yields of transfected cells. Nonetheless, a notable fraction of cells were successfully transfected via electroporation. Distinct differences emerged upon analyzing the individual transfections. CSFV-Ubi-C_1512_A-Δcore and CSFV-Ubi-C_1512_A-Δcore-NS2-3-N_2177_Y exhibited replication primarily in single cells and doublets, whereas groups and a higher number of E2-positive cells appeared in CSFV-Ubi-C_1512_A-Δcore-NS3-N_2177_Y and CSFV-Ubi-C_1512_A-Δcore-NS2-3-N_2177_Y-NS3-N_2177_Y. The contrast became even more evident post-inoculation of naïve cells using the supernatants of the transfected cells (virus passage). CSFV-Ubi-C_1512_A-Δcore and CSFV-Ubi-C_1512_A-Δcore-NS2-3-N_2177_Y completely failed to produce infectious particles. However, CSFV-Ubi-C_1512_A-Δcore-NS3-N_2177_Y and CSFV-Ubi-C_1512_A-Δcore-NS2-3-N_2177_Y-NS3-N_2177_Y yielded similar titers with 2.2 × 10^3^ and 1.3 × 10^3^ TCID_50_/mL, respectively. Like the parent virus (CSFV-Ubi), both viruses showed cytopathogenic properties and multiplied with plaque formation (Fig. 12).

The titers of these viruses (Table 6) were comparatively low and about one log level below those of CSFV-Ubi without core deletion (27). However, the difference in titer nicely corresponds to the difference observed between the noncytopathic wtCSFV and the coreless CSFV bearing the compensatory NS3 mutation N_2177_Y (25).

**TABLE 6:** Packaging of cytopathogenic CSFVs with genomic duplications bearing uncleavable NS2-3 and a free NS3 moiety

Cytopathogenic CSFVs	Progeny virus production
CSFV-Ubi-C_1512_A-Δcore	n.d.^*a*^
CSFV-Ubi-C_1512_A-Δcore-NS2-3-N_2177_Y	n.d.^*a*^
CSFV-Ubi-C_1512_A-Δcore-NS3-N_2177_Y	2.2 × 10^3^ TCID_50_/mL
CSFV-Ubi-C_1512_A-Δcore-NS2-3-N_2177_Y-NS3-N_2177_Y	1.3 × 10^3^ TCID_50_/mL

^*a*^ n.d. means not detected.

**Fig 13:**
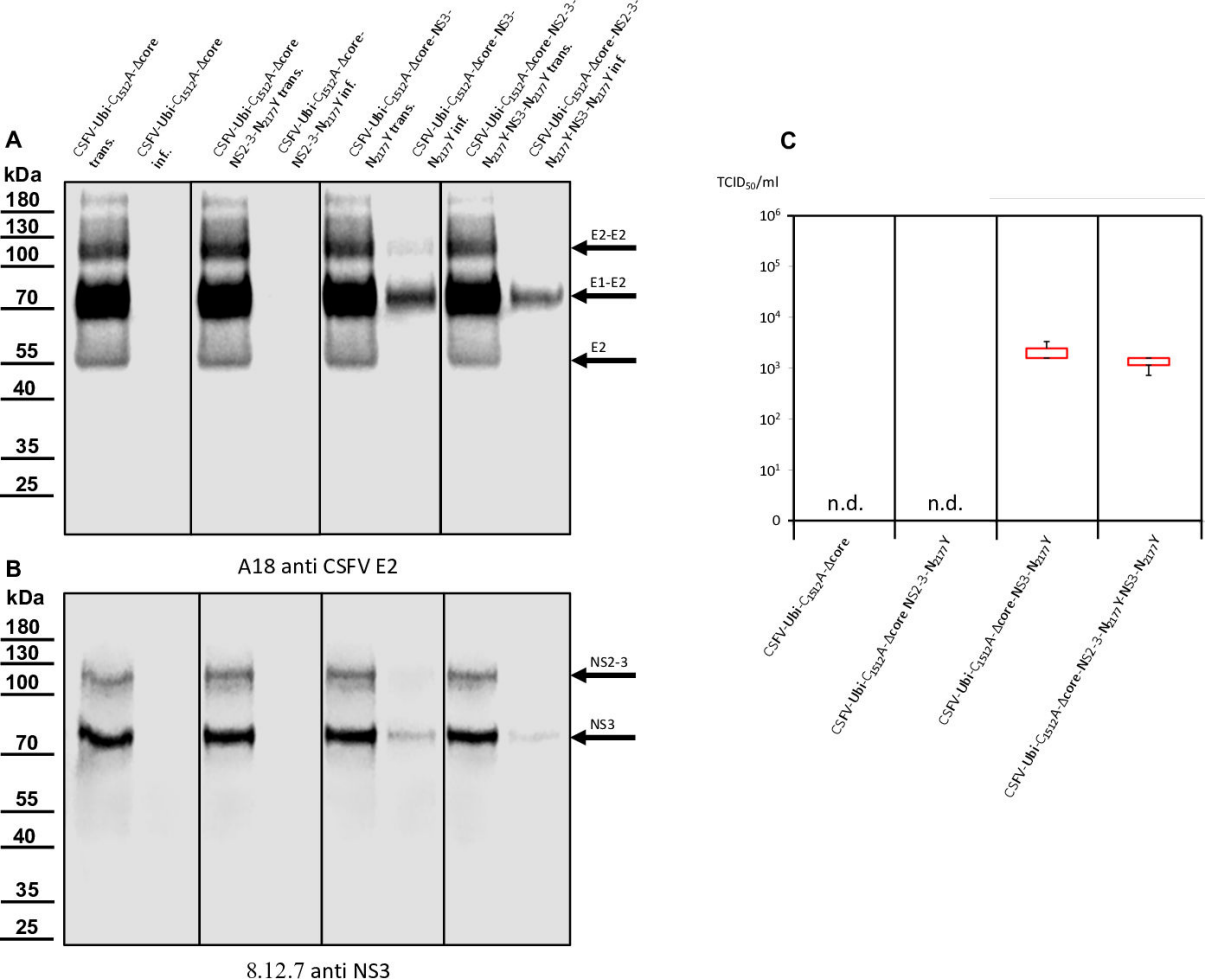
Western blot analyses were conducted on cells transfected or infected with cytopathogenic CSFV-Ubi strains. (**A**) Expression of E2, evident through strong bands at 80 kDa and additional bands at 55 as well as 100 kDa, was consistently observed post-transfection. However, in infection experiments, E2 expression was solely visible in the case of CSFV-Ubi-C_1512_A-Δcore-NS3-N_2177_Y and CSFV-Ubi-C_1512_A-Δcore-NS2-3-N_2177_Y-NS3-N_2177_Y. (**B**) Simultaneous expression of NS2-3 and NS3, marked by bands at 125 and 80 kDa, was evident after transfection of all coreless CSFV-Ubi clones. However, NS2-3 and NS3 expressions were solely detectable in case of infection with CSFV-Ubi-C_1512_A-Δcore-NS3-N_2177_Y and CSFV-Ubi-C_1512_A-Δcore-NS2-3-N_2177_Y-NS3-N_2177_Y. (**C**) It was possible to detect progeny virus production only in the case of the two genomes with the compensatory mutation in mature NS3 presenting titers that exceeded 1 × 10^3^ TCID_50_/mL.

Western blot analyses utilizing E2-specific antibodies confirmed strong protein expression of all the cytopathic genomes, with a dominant signal at 80 kDa. Notably, the first two constructs did not exhibit progeny virus production, whereas CSFV-Ubi-C_1512_A-Δcore-NS3-N_2177_Y and CSFV-Ubi-C_1512_A-Δcore-NS2-3-N_2177_Y-NS3-N_2177_Y did (Fig. 13A). Successful transfection and particle formation were also evident in the case of CSFV-Ubi-C_1512_A-Δcore-NS3-N_2177_Y and CSFV-Ubi-C_1512_A-Δcore-NS2-3-N_2177_Y-NS3-N_2177_Y when using the NS3-specific antibody. Simultaneous expression of NS2-3 and mature NS3, characteristic of cytopathic viruses (Fig. 13B) was visible in all these genomes after RNA transfection. However, only the constructs carrying the compensatory mutation N_2177_Y in mature NS3 managed to produce progeny virus, highlighting that mature NS3 orchestrates packaging in the absence of core protein. This finding fits closely with the results of our *trans-*packaging assays but is surprising in many ways. It challenges the conventional understanding of pestiviruses with duplicated NS3 coding sequences, suggesting a functional interconnection rather than strict separation between the packaging and replication modules within the genome. Although the NS2-3-4A cassette following the structural proteins was previously assigned to the packaging module, the NS3-NS5B cassette behind the additional processing signal should form an independent functional replication module at the C-terminus of the polyprotein. Our data suggest that the mature NS3 or even the entire replication module is actively involved in the assembly of virions.

### CSFV does not *trans*-package DIs of Linda pestivirus and BVDV-1

**Fig 14:**
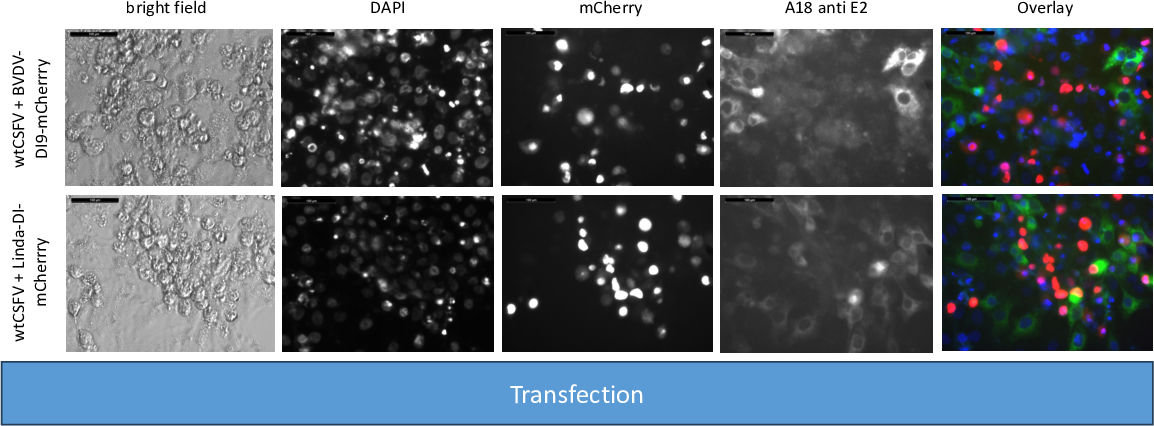
Transfection of SK-6 cells with wtCSFV and DIs of BVDV-1 and Linda pestivirus. Synthetic RNA of wtCSFV and the reporter DIs BVDV-DI9-mCherry and Linda-DI-mCherry were transfected via electroporation. After 24 hours, the cells were fixed and immunostained for the E2 protein. Nuclei were counterstained with DAPI, and fluorescence was captured using a monochromatic camera at 20-fold magnification. To enhance visualization, the images were overlaid with DAPI in blue, mCherry in red, and E2 in green. A size marker of 100 µm length was included in the upper left corner for reference.

A pivotal role of the mature NS3 encoded in *cis* for genome packaging was clearly demonstrated in CSFV. We aimed to extend these findings to other pestiviruses to explore potential similarities in packaging mechanisms. However, coreless strains have only been identified for CSFV, despite the highly conserved nature of NS3 among classical pestiviruses. To investigate amino acids that functionally compensate for the lack of a core, we tested the ability of CSFV to *trans*-package replicative subgenomes from BVDV-1 and Linda pestivirus. For these experiments, similar reporter constructs were generated, featuring a mCherry reporter following amino acid 15 of the N^pro^. Replication and reporter gene expression were confirmed after co-transfection with CSFVwt (Fig. 14). Notably, the BVDV-1 DI9 subgenome exhibited stronger replication compared with the engineered Linda pestivirus DI or the CSFV-DIs, which also lacked the structural protein region but were derived from a wild-type genome.

**Fig 15:**
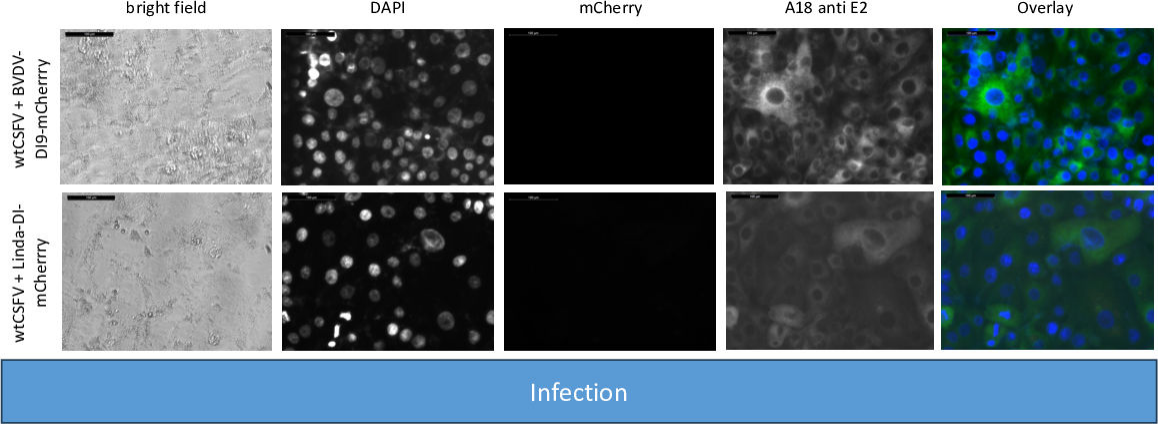
Infecting SK-6 cells with cell culture supernatants from wtCSFV and BVDV-DI9-mCherry as well as wtCSFV and Linda-DI-mCherry transfected cells. Naïve SK-6 cells were inoculated with 100 µL of sterile filtered cell culture supernatant harvested 48 hours post-transfection. After a 48 hour incubation period, the cells were fixed and immunostained for the E2 protein, with nuclei counterstained using DAPI. Fluorescence was captured via a monochromatic camera at 20-fold magnification. For enhanced visualization, the images were overlaid, presenting DAPI in blue, mCherry in red, and E2 in green. No mCherry signals were found. A size marker, 100 µm in length, was incorporated in the upper left corner for reference.

Inoculating naïve SK-6 cells with supernatants from the co-transfections revealed that neither subgenome was packaged by the CSFV helper virus (Fig. 15). Given the significant genetic divergence between Linda pestivirus and CSFV (only approximately 50% amino acid identity), such a result was not unexpected due to likely incompatibilities between the protein. However, the lack of *trans*-packaging of the BVDV-1 DI9 subgenome was surprising, as CSFV (Alfort-Tübingen strain) and BVDV-1 (DI9 closely matching the full genome of BVDV-1 CP7) share over 71% amino acid identity. This outcome underscores the high specificity of pestiviral packaging and highlights the critical role of *cis*-acting elements. These negative results leave open the question of whether important *cis*-active elements are effective in the untranslated RNA molecule or within the translated proteins.

## DISCUSSION

Within the *Flavivirus*, *Hepacivirus*, and *Pestivirus* genera, the viral nucleic acids are encapsidated by a positively charged core protein, forming a nucleoprotein complex or nucleocapsid. Conversely, the *Pegivirus* genus lacks a comparable capsid protein, suggesting a degree of flexibility in RNA packaging and virion assembly requirements (39). Assembly of infectious particles occurs at the membranes of the endoplasmic reticulum (ER), where ribonucleoprotein complexes are enclosed within a viral envelope containing genus-specific glycoproteins: prM and E for *Flavivirus*, E1 and E2 for *Hepacivirus* and *Pegivirus*, or E^rns^, E1, and E2 for *Pestivirus*. The exact molecular mechanisms that control the selective packaging of viral RNA have not yet been clarified, although the presence of a specific packaging signal within the RNA sequence seems unlikely (1). For non-enveloped plus-stranded RNA viruses, on the other hand, direct coupling of replication and encapsidation of RNA was demonstrated using *trans-*complementation, providing an encapsidation model that ensures high packaging specificity (40). Research into Flavivirus packaging has yielded a comprehensive model, wherein the core plays a central role in driving encapsidation by acting as an RNA-binding protein and interacting with the non-structural proteins (41, 42). Conversely, budding, the process of membrane acquisition, is primarily mediated by the glycoproteins and even takes place in the absence of nucleocapsids (43). Even naturally infected cells release subviral particles that contain prM-E in the envelope but lack nucleocapsids and nucleic acids, suggesting a relatively unspecific budding process. *Trans-*packaging experiments using replicative flavivirus subgenomes and alphavirus expression systems have highlighted packaging specificity for non-translated regions (NTRs) together with the non-structural protein region of flaviviruses (44). Similar observations extend to pestiviruses, where helper viruses complement structural protein deficiencies of replicative subgenomes (45, 46), and hepaciviruses, where replicons could be *trans*-complemented by structural proteins expressed through DNA transfection and nuclear expression of virion structural components (47). These findings underscore a mechanism of RNA encapsidation driven by core protein and non-structural protein interaction, with glycoproteins readily available at budding sites waiting to pack the nucleocapsids.

However, the specific role of non-structural proteins in recruiting genomic RNA for packaging in the virion envelope remains to be fully elucidated. Mutational analyses across various flaviviruses have revealed the direct involvement of NS2A and NS3 in RNA packaging. Packaging deficiencies observed in NS2A mutants could be rescued by *trans*-expression, suggesting a similar level of accessibility as observed for the structural proteins (48). Remarkably, certain NS2A mutations hindering infectious particle production could be compensated by second-site mutations in the NS3 helicase domain (49). However, for Kunjin virus, packaging deficient NS3 mutants proved non-compensable by *trans*-expression, highlighting a pivotal *cis*-active role of NS3 in RNA recruitment for genome encapsidation and/or budding (50). In contrast, studies on yellow fever virus (YFV) *trans*-complementation indicated the possibility of NS3 *trans*-complementation, albeit with low efficiency (51). Specific amino acid mutations in NS3 could be assigned to packaging defects, although they had little or no effect on the replicative capacity of the genomes. The conserved tryptophan residue W_349_ in the NS3 helicase was found to be crucial for YFV infectious particle production, implicating distinct functions of this domain in the assembly process (52). In the case of Dengue fever virus (DENV), residues W_5_ in the protease domain of NS3, I_178_ in the protease-helicase linker region, and N_576_ in the NS3 helicase domain were found to be dispensable for RNA replication but pivotal for infectious particle production (53). Similarly, the E_530_ residue in the HCV NS3 helicase was identified as pivotal for virus particle formation without impacting viral genome replication (54). In summary, flaviviral and hepaciviral NS3 appear to play a complex, yet incompletely understood role as a gatekeeper in viral morphogenesis.

Pestivirus genome packaging must be distinguished from the processes in the other genera due to obvious differences. Numerous studies delve into the RNA packaging mechanisms of pestiviruses, as these can provide unique insights due to the existence of distinguishable biotypes. Non-cytopathogenic and cytopathogenic BVDVs have been extensively studied, revealing a temporal regulation of the NS2-3-4A precursor processing linked to the availability of the cellular co-factor DNAJC14. This co-factor activates the NS2 auto-protease and mediates the cleavage between NS2 and NS3 (55). Hence, the processing of NS2-3 serves as a switch between early viral RNA replication and later virion assembly (16). During the early phases of BVDV infection, only free NS3, the fully processed major viral protease, is detectable. This suggests that the processing speed and the balance between polyprotein precursors and mature non-structural proteins play crucial roles in determining the fate of RNA molecules, whether they contribute to translation and replication or are sent to assembly and budding. Moreover, it has been demonstrated across various pestiviruses that co-expression of the uncleaved NS2-3 precursor in conjunction with NS4A is essential for RNA packaging (17). However, the indispensable role of uncleaved NS2-3 precursors in RNA packaging of pestiviruses was challenged by synthetic BVDV and CSFV strains (18, 19). These strains, engineered with NS2 and NS3 separated by an amber codon and an IRES inserted in between, were able to form particles in the absence of NS2-3 precursors, following multiple passages and accumulation of single mutations.

**Fig 16:**
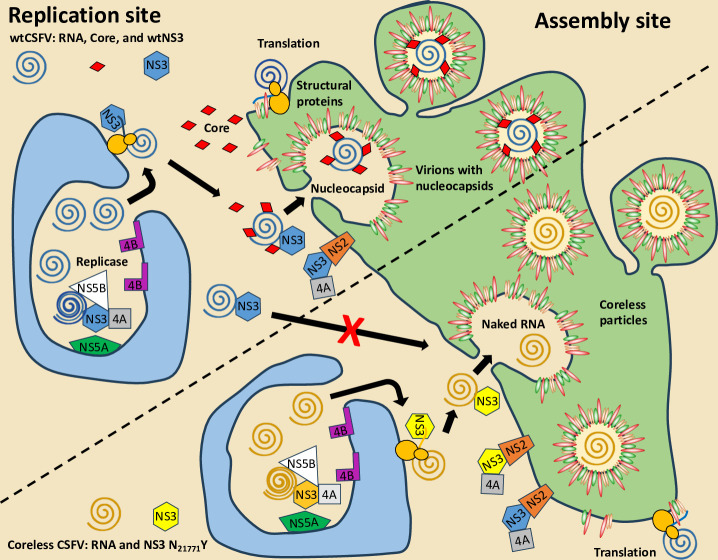
Model of genome packaging in wild-type and core-free CSFVs. The viral RNA is replicated from plus-minus strand intermediates in membranous invaginations of the endoplasmic reticulum (ER), likely double-membrane vesicles. The replication machinery consists of the non-structural proteins NS3, NS4A, NS4B, NS5A, and NS5B. NS4B is responsible for the acquisition of membranes, whereas the other non-structural proteins are actively involved in RNA replication, with the RNA-dependent RNA polymerase NS5B playing a crucial role. No differences in RNA replication were observed between core-coding and coreless CSFV genomes. The replicated genomic plus strands are transported outside these compartments and translated at ER membranes. The newly synthesized NS3 or a larger complex containing NS3 maintains contact with the coding RNA molecule (*cis*-activity). Genome-bound NS3 loads core protein onto the RNA and directs the nucleocapsid to the assembly site, where membrane acquisition occurs at pre-formed pits. Only mutant NS3 (NS3-N_2177_Y) can envelop the RNA at assembly sites in the absence of the core. Uncleaved NS2-3-4A precursor molecules, which are also essential for the packaging process, are available for packaging genomes, regardless of the N_2177_Y mutation. The pool of glycoproteins in host cell membranes is also freely available. Finally, the virions are released by cellular exocytosis. Hence, RNA packaging in CSFV is controlled by the mature *cis*-active NS3.

In this study, we investigated the role of NS3 in virion assembly in even more detail. A finding from previous research was the identification of a coreless CSFV strain, which revealed that specific mutations in the third domain of the NS3 helicase can compensate for the absence of core protein, resulting in the production of core-free CSFV particles (25). Even in the presence of core protein, these mutations prevented the core from being incorporated into virions. Here, we addressed the question of whether the mutations compensating for core protein deficiency act in the uncleaved NS2-3 precursor or the mature NS3. We employed a traditional *trans*-complementation assay involving NS3-expressing packaging defective subgenomes and NS2-3-expressing helper viruses. Leveraging the coreless CSFV strain as a tool for further elucidating NS3-mediated packaging mechanisms, our investigation revealed that mature NS3 facilitates genome packaging in a *cis*-dependent manner. It became evident that the mutant NS3 molecules exclusively acted on their own RNA, failing to induce *trans*-packaging of genomes harboring the wild-type NS3 sequence (Fig. 16). Moreover, *trans*-packaging experiments unveiled the efficacy of these mutations in both the cleavable NS2-3 of the helper viruses and the mature NS3 of the subgenomes. Although previous knowledge indicated that the functions of NS3 in the pestiviral replicase could not be complemented by *trans*-expression, existing data had suggested effective *trans*-complementation of pestiviral NS2-3-4A in particle formation (17).

For the first time, our findings shed light on a previously unrecognized *cis* function of mature NS3 in pestiviral genome packaging. We found that the mature NS3 acted as a gatekeeper by specifically forwarding only those RNA molecules to the molecular packaging machinery that encoded it. In contrast, the structural proteins were freely available within the infected cells and served to package whatever was being passed on by a functional NS3. To investigate whether *cis*-packaging could also involve uncleaved NS2-3 precursors, we utilized a recently described CSFV clone containing duplicated NS3 coding sequences (27). In coreless CSFVs with duplicated NS3s, the mutation also compensated for core deficiency and restored particle assembly when present in mature NS3. However, it failed to induce particle formation when only present in the uncleavable NS2-3 precursor. This underscores the indispensable *cis* role of mature NS3 in viral RNA packaging in the case of coreless CSFV. This process is very interesting because coupling RNA packaging to distinct functions of direct translation products could serve as an indirect proofreading mechanism, ensuring encapsidation and transmission of only those RNA molecules capable of expressing the functional protein. This could represent an indirect quality control measure and minimize the transmission of defective RNA molecules that are constantly produced by the error-prone RNA-dependent RNA polymerase. A comparable mechanism was observed in the flock house virus (FHV), where translation of capsid proteins in *cis* is essential for genome encapsidation, suggesting that it might represent a common packaging quality control system for many RNA viruses (56, 57). However, the involvement of NS3 in *cis* in pestivirus assembly is likely more complex, given that the NS3 protein is not found within virions. A potential model could include co-translational binding of proteins to the RNA and direct packaging. Yet to be defined protein-protein interactions involving the core protein could mediate and regulate this process in a concentration-dependent manner. The observed single amino acid exchanges in the NS3 helicase domain 3, which compensate for core deficiency, likely disrupt these interactions, resulting in a core loading defect and subsequent coreless particle assembly.

The role of NS3 in the pestiviral packaging process appears to be more intricate than originally assumed. Both NS2-3-4A and mature NS3 exhibit essential functions, likely mediated through protein-protein and protein-RNA interactions. A protein surface at the tip of the third subdomain of the NS3 helicase, containing amino acid N_2177_, seems to regulate nucleocapsid loading to newly budding virions. Since different viral proteins contribute to virion assembly and RNA packaging, the mature NS3 probably operates as part of a larger complex. NS5B of BVDV is known to play a crucial role in particle assembly, since mutations in its C-terminus can facilitate active replication but impede particle formation entirely (58). In the case of different RNA viruses, only actively replicated RNA was packaged, suggesting that packaging is restricted to progeny RNA strands emerging from replication complexes (59).

In 2008, Liang et al. explored the *trans*-complementation of pestiviral reporter DIs packaged using vaccinia virus-encoded helper viruses (45). By applying N- and C-terminal reciprocal truncations, they discovered that pseudo-particle packaging requires p7 and the contiguous NS2-3-4A precursor. Although nonstructural proteins such as p7, NS4B, NS5A, and NS5B could be expressed either in *cis* or in *trans* for particle production, they highlighted that replication, mainly reliant on mature NS3 expression, is crucial for efficient particle formation. Interestingly, they reported that non-replicative RNAs could also be packaged, evidenced solely by the detection of E2 in cell culture supernatant. However, with current knowledge confirming that structural protein expression alone can produce nucleocapsid-free pseudo virions, this conclusion now warrants re-evaluation. Last year, Fellenberg et al. investigated NS4A’s role in replication and particle formation (60). They discovered that mutations in NS4A that allowed replication, but impaired particle formation could be compensated by second-site mutations in NS2 and NS3. Specifically, mutations in the NS4A linker region and C-terminus hindered particle formation but could be restored through NS2-3-4A *trans*-complementation or compensatory mutations. Although the gain-of-function study emphasized the NS2-3-4A’s role as a packaging scaffold that toggles between replication and packaging, it left questions about mature NS3’s functions.

In this study, we explored gain of function of mature NS3 for the first time using coreless CSFV strains. We demonstrated that mature NS3 and likely the entire pestiviral replicase are crucial for genome packaging. This suggests that mature NS3, correct nucleocapsid assembly, and likely the identity of the other non-structural proteins are key to packaging specificity. Our findings support a model, in which genome replication and assembly are linked for high efficiency and specificity. In conclusion, our study emphasizes that mature NS3, rather than the uncleaved NS2-3 precursor, determines the immediate fate of the viral RNA molecules in coreless CSFV. Future studies must show whether similar processes also apply to the packaging of the RNA in the presence of the core protein and how NS3 facilitates nucleocapsid formation.

## Data Availability

The authors confirm that all data supporting the findings of this study are available within the article and its supplementary materials. Further details on the procedure of all experiments can be obtained from the corresponding author.

## SUPPLEMENTAL MATERIAL

Supplemental legends jvi.01209-24-s0001.docx Legends for Movies S1 to S3.

Movie S1 jvi.01209-24-s0002.mp4 Live-cell imaging of wtCSFV/CSFV-DI-mCherry infected SK-6 cells.

Movie S2 jvi.01209-24-s0003.mp4 Live-cell imaging of wtCSFV/CSFV-DI-N2177Y-mCherry infected SK-6 cells.

Movie S3 jvi.01209-24-s0004.mp4 Live-cell imaging of CSFV-Δcore-N2177Y/CSFV-DI-N2177Y-mCherry infected SK-6 cells.

ASM does not own the copyrights to Supplemental Material that may be linked to, or accessed through, an article. The authors have granted ASM a non-exclusive, world-wide license to publish the Supplemental Material files. Please contact the corresponding author directly for reuse.

## References

[1] Simmonds P, Becher P, Bukh J, Gould EA, Meyers G, Monath T, Muerhoff S, Pletnev A, Rico-Hesse R, Smith DB, Stapleton JT, Ictv Report Consortium. 2017. ICTV virus taxonomy profile: Flaviviridae. J Gen Virol 98:2–3. doi:10.1099/jgv.0.000672 28218572 PMC5370391

[2] Smith DB, Meyers G, Bukh J, Gould EA, Monath T, Scott Muerhoff A, Pletnev A, Rico-Hesse R, Stapleton JT, Simmonds P, Becher P. 2017. Proposed revision to the taxonomy of the genus Pestivirus, family Flaviviridae. J Gen Virol 98:2106–2112. doi:10.1099/jgv.0.000873 28786787 PMC5656787

[3] Wu Z, Liu B, Du J, Zhang J, Lu L, Zhu G, Han Y, Su H, Yang L, Zhang S, Liu Q, Jin Q. 2018. Discovery of diverse rodent and bat pestiviruses with distinct genomic and phylogenetic characteristics in several Chinese provinces. Front Microbiol 9:2562. doi:10.3389/fmicb.2018.02562 30405596 PMC6207626

[4] Gao WH, Lin XD, Chen YM, Xie CG, Tan ZZ, Zhou JJ, Chen S, Holmes EC, Zhang YZ. 2020. Newly identified viral genomes in pangolins with fatal disease. Virus Evol 6:veaa020. doi:10.1093/ve/veaa020 32296543 PMC7151644

[5] Jo WK, van Elk C, van de Bildt M, van Run P, Petry M, Jesse ST, Jung K, Ludlow M, Kuiken T, Osterhaus A. 2019. An evolutionary divergent pestivirus lacking the N^pro^ gene systemically infects a whale species. Emerg Microbes Infect 8:1383–1392. doi:10.1080/22221751.2019.1664940 31526243 PMC6758615

[6] Bartenschlager R, Ahlborn-Laake L, Yasargil K, Mous J, Jacobsen H. 1995. Substrate determinants for cleavage in cis and in trans by the hepatitis C virus NS3 proteinase. J Virol 69:198–205. doi:10.1128/JVI.69.1.198-205.1995 7983710 PMC188564

[7] De Francesco R, Steinkühler C. 2000. Structure and function of the hepatitis C virus NS3-NS4A serine proteinase. Curr Top Microbiol Immunol 242:149–169. doi:10.1007/978-3-642-59605-6_8 10592660

[8] Hilgenfeld R, Lei J, Zhang L. 2018. The structure of the Zika virus protease, NS2B/NS3^pro^. Adv Exp Med Biol 1062:131–145. doi:10.1007/978-981-10-8727-1_10 29845530

[9] Borowski P, Niebuhr A, Schmitz H, Hosmane RS, Bretner M, Siwecka MA, Kulikowski T. 2002. NTPase/helicase of Flaviviridae: inhibitors and inhibition of the enzyme. Acta Biochim Pol 49:597–614. 12422230

[10] Yerukhimovich MM, Marohnic CC, Frick DN. 2018. Role of the conserved DECH-box cysteine in coupling hepatitis C virus helicase-catalyzed ATP hydrolysis to RNA unwinding. Biochemistry 57:6247–6255. doi:10.1021/acs.biochem.8b00796 30281972 PMC8532174

[11] Tortorici MA, Duquerroy S, Kwok J, Vonrhein C, Perez J, Lamp B, Bricogne G, Rümenapf T, Vachette P, Rey FA. 2015. X-ray structure of the pestivirus NS3 helicase and its conformation in solution. J Virol 89:4356–4371. doi:10.1128/JVI.03165-14 25653438 PMC4442355

[12] Lu H, Zhan Y, Li X, Bai X, Yuan F, Ma L, Wang X, Xie M, Wu W, Chen Z. 2021. Novel insights into the function of an N-terminal region of DENV2 NS4B for the optimal helicase activity of NS3. Virus Res 295:198318. doi:10.1016/j.virusres.2021.198318 33485995

[13] Tai CL, Chi WK, Chen DS, Hwang LH. 1996. The helicase activity associated with hepatitis C virus nonstructural protein 3 (NS3). J Virol 70:8477–8484. doi:10.1128/JVI.70.12.8477-8484.1996 8970970 PMC190938

[14] Isken O, Postel A, Bruhn B, Lattwein E, Becher P, Tautz N. 2019. CRISPR/Cas9-mediated knockout of DNAJC14 verifies this chaperone as a pivotal host factor for RNA replication of pestiviruses. J Virol 93:e01714-18. doi:10.1128/JVI.01714-18 30518653 PMC6384085

[15] Lackner T, Müller A, Pankraz A, Becher P, Thiel HJ, Gorbalenya AE, Tautz N. 2004. Temporal modulation of an autoprotease is crucial for replication and pathogenicity of an RNA virus. J Virol 78:10765–10775. doi:10.1128/JVI.78.19.10765-10775.2004 15367643 PMC516412

[16] Agapov EV, Murray CL, Frolov I, Qu L, Myers TM, Rice CM. 2004. Uncleaved NS2-3 is required for production of infectious bovine viral diarrhea virus. J Virol 78:2414–2425. doi:10.1128/jvi.78.5.2414-2425.2004 14963137 PMC369244

[17] Moulin HR, Seuberlich T, Bauhofer O, Bennett LC, Tratschin JD, Hofmann MA, Ruggli N. 2007. Nonstructural proteins NS2-3 and NS4A of classical swine fever virus: essential features for infectious particle formation. Virology (Auckl) 365:376–389. doi:10.1016/j.virol.2007.03.056 17482232

[18] Klemens O, Dubrau D, Tautz N. 2015. Characterization of the determinants of NS2-3-independent virion morphogenesis of pestiviruses. J Virol 89:11668–11680. doi:10.1128/JVI.01646-15 26355097 PMC4645674

[19] Dubrau D, Schwindt S, Klemens O, Bischoff H, Tautz N. 2019. Determination of critical requirements for classical swine fever virus NS2-3-independent virion formation. J Virol 93:e00679-19. doi:10.1128/JVI.00679-19 31292243 PMC6714812

[20] Kümmerer BM, Tautz N, Becher P, Thiel H, Meyers G. 2000. The genetic basis for cytopathogenicity of pestiviruses. Vet Microbiol 77:117–128. doi:10.1016/s0378-1135(00)00268-6 11042405

[21] Lamp B, Riedel C, Roman-Sosa G, Heimann M, Jacobi S, Becher P, Thiel H-J, Rümenapf T. 2011. Biosynthesis of classical swine fever virus nonstructural proteins. J Virol 85:3607–3620. doi:10.1128/JVI.02206-10 21270154 PMC3067844

[22] Yamane D, Kato K, Tohya Y, Akashi H. 2006. The double-stranded RNA-induced apoptosis pathway is involved in the cytopathogenicity of cytopathogenic Bovine viral diarrhea virus. J Gen Virol 87:2961–2970. doi:10.1099/vir.0.81820-0 16963755

[23] Khromykh AA, Westaway EG. 1997. Subgenomic replicons of the flavivirus kunjin: construction and applications. J Virol 71:1497–1505. doi:10.1128/JVI.71.2.1497-1505.1997 8995675 PMC191206

[24] Pang X, Guo Y, Zhou Y, Fu W, Gu X. 2014. Highly efficient production of a dengue pseudoinfectious virus. Vaccine (Auckl) 32:3854–3860. doi:10.1016/j.vaccine.2014.03.091 PMC4083744 24797700

[25] Riedel C, Lamp B, Heimann M, König M, Blome S, Moennig V, Schüttler C, Thiel HJ, Rümenapf T. 2012. The core protein of classical Swine Fever virus is dispensable for virus propagation in vitro. PLoS Pathog 8:e1002598. doi:10.1371/journal.ppat.1002598 22457622 PMC3310793

[26] Moser C, Stettler P, Tratschin JD, Hofmann MA. 1999. Cytopathogenic and noncytopathogenic RNA replicons of classical swine fever virus. J Virol 73:7787–7794. doi:10.1128/JVI.73.9.7787-7794.1999 10438869 PMC104306

[27] Reuscher CM, Schmidt L, Netsch A, Lamp B. 2021. Characterization of a cytopathogenic reporter CSFV. Viruses 13:1209. doi:10.3390/v13071209 34201706 PMC8310069

[28] Kasza L, Shadduck JA, Christofinis GJ. 1972. Establishment, viral susceptibility and biological characteristics of a swine kidney cell line SK-6. Res Vet Sci 13:46–51. doi:10.1016/S0034-5288(18)34087-6 4336054

[29] Rümenapf T, Meyers G, Stark R, Thiel HJ. 1991. Molecular characterization of hog cholera virus. Arch Virol Suppl 3:7–18. doi:10.1007/978-3-7091-9153-8_2 9210921

[30] Meyers G, Tautz N, Becher P, Thiel HJ, Kümmerer BM. 1996. Recovery of cytopathogenic and noncytopathogenic bovine viral diarrhea viruses from cDNA constructs. J Virol 70:8606–8613. doi:10.1128/JVI.70.12.8606-8613.1996 8970985 PMC190953

[31] Tautz N, Thiel HJ, Dubovi EJ, Meyers G. 1994. Pathogenesis of mucosal disease: a cytopathogenic pestivirus generated by an internal deletion. J Virol 68:3289–3297. doi:10.1128/JVI.68.5.3289-3297.1994 8151789 PMC236819

[32] Reuscher CM, Seitz K, Schwarz L, Geranio F, Isken O, Raigel M, Huber T, Barth S, Riedel C, Netsch A, Zimmer K, Rümenapf T, Tautz N, Lamp B. 2022. DNAJC14-independent replication of the atypical porcine pestivirus. J Virol 96:e0198021. doi:10.1128/jvi.01980-21 35852352 PMC9364808

[33] Corapi WV, Donis RO, Dubovi EJ. 1988. Monoclonal antibody analyses of cytopathic and noncytopathic viruses from fatal bovine viral diarrhea virus infections. J Virol 62:2823–2827. doi:10.1128/JVI.62.8.2823-2827.1988 2455820 PMC253717

[34] Lamp B, Schwarz L, Högler S, Riedel C, Sinn L, Rebel-Bauder B, Weissenböck H, Ladinig A, Rümenapf T. 2017. Novel pestivirus species in pigs, Austria, 2015. Emerg Infect Dis 23:1176–1179. doi:10.3201/eid2307.170163 28628456 PMC5512468

[35] Schägger H, von Jagow G. 1987. Tricine-sodium dodecyl sulfate-polyacrylamide gel electrophoresis for the separation of proteins in the range from 1 to 100 kDa. Anal Biochem 166:368–379. doi:10.1016/0003-2697(87)90587-2 2449095

[36] Li Y, Shen L, Li C, Huang J, Zhao B, Sun Y, Li S, Luo Y, Qiu HJ. 2014. Visualization of the N^pro^ protein in living cells using biarsenically labeling tetracysteine-tagged classical swine fever virus. Virus Res 189:67–74. doi:10.1016/j.virusres.2014.04.018 24815879

[37] Li Y, Wang X, Sun Y, Li L-F, Zhang L, Li S, Luo Y, Qiu H-J. 2016. Generation and evaluation of a chimeric classical swine fever virus expressing a visible marker gene. Arch Virol 161:563–571. doi:10.1007/s00705-015-2693-7 26614259

[38] Tautz N, Meyers G, Thiel HJ. 1993. Processing of poly-ubiquitin in the polyprotein of an RNA virus. Virology (Auckl) 197:74–85. doi:10.1006/viro.1993.1568 8212597

[39] Stapleton JT, Foung S, Muerhoff AS, Bukh J, Simmonds P. 2011. The GB viruses: a review and proposed classification of GBV-A, GBV-C (HGV), and GBV-D in genus Pegivirus within the family Flaviviridae. J Gen Virol 92:233–246. doi:10.1099/vir.0.027490-0 21084497 PMC3081076

[40] Nugent CI, Johnson KL, Sarnow P, Kirkegaard K. 1999. Functional coupling between replication and packaging of poliovirus replicon RNA. J Virol 73:427–435. doi:10.1128/JVI.73.1.427-435.1999 9847348 PMC103849

[41] Jablunovsky A, Jose J. 2024. The dynamic landscape of capsid proteins and viral RNA interactions in flavivirus genome packaging and virus assembly. Pathogens 13:120. doi:10.3390/pathogens13020120 38392858 PMC10893219

[42] Zayas M, Long G, Madan V, Bartenschlager R. 2016. Coordination of hepatitis C virus assembly by distinct regulatory regions in nonstructural protein 5A. PLoS Pathog 12:e1005376. doi:10.1371/journal.ppat.1005376 26727512 PMC4699712

[43] Konishi E, Fujii A, Mason PW. 2001. Generation and characterization of a mammalian cell line continuously expressing Japanese encephalitis virus subviral particles. J Virol 75:2204–2212. doi:10.1128/JVI.75.5.2204-2212.2001 11160724 PMC114804

[44] Khromykh AA, Varnavski AN, Westaway EG. 1998. Encapsidation of the flavivirus kunjin replicon RNA by using a complementation system providing Kunjin virus structural proteins in trans. J Virol 72:5967–5977. doi:10.1128/JVI.72.7.5967-5977.1998 9621059 PMC110401

[45] Liang D, Chen L, Ansari IH, Gil L, Topliff CL, Kelling CL, Donis RO. 2009. A replicon trans-packaging system reveals the requirement of nonstructural proteins for the assembly of bovine viral diarrhea virus (BVDV) virion. Virol Auckl 387:331–340. doi:10.1016/j.virol.2009.02.019 19327808

[46] Behrens SE, Grassmann CW, Thiel HJ, Meyers G, Tautz N. 1998. Characterization of an autonomous subgenomic pestivirus RNA replicon. J Virol 72:2364–2372. doi:10.1128/JVI.72.3.2364-2372.1998 9499097 PMC109536

[47] Adair R, Patel AH, Corless L, Griffin S, Rowlands DJ, McCormick CJ. 2009. Expression of hepatitis C virus (HCV) structural proteins in trans facilitates encapsidation and transmission of HCV subgenomic RNA. J Gen Virol 90:833–842. doi:10.1099/vir.2008.006049-0 19223490

[48] Xie X, Zou J, Puttikhunt C, Yuan Z, Shi PY. 2015. Two distinct sets of NS2A molecules are responsible for dengue virus RNA synthesis and virion assembly. J Virol 89:1298–1313. doi:10.1128/JVI.02882-14 25392211 PMC4300643

[49] Voßmann S, Wieseler J, Kerber R, Kümmerer BM. 2015. A basic cluster in the N terminus of yellow fever virus NS2A contributes to infectious particle production. J Virol 89:4951–4965. doi:10.1128/JVI.03351-14 25694595 PMC4403467

[50] Pijlman GP, Kondratieva N, Khromykh AA. 2006. Translation of the flavivirus kunjin NS3 gene in cis but not its RNA sequence or secondary structure is essential for efficient RNA packaging. J Virol 80:11255–11264. doi:10.1128/JVI.01559-06 16971441 PMC1642170

[51] Jones CT, Patkar CG, Kuhn RJ. 2005. Construction and applications of yellow fever virus replicons. Virology (Auckl) 331:247–259. doi:10.1016/j.virol.2004.10.034 15629769

[52] Patkar CG, Kuhn RJ. 2008. Yellow Fever virus NS3 plays an essential role in virus assembly independent of its known enzymatic functions. J Virol 82:3342–3352. doi:10.1128/JVI.02447-07 18199634 PMC2268495

[53] Gebhard LG, Iglesias NG, Byk LA, Filomatori CV, De Maio FA, Gamarnik AV. 2016. A proline-rich N-terminal region of the dengue virus NS3 is crucial for infectious particle production. J Virol 90:5451–5461. doi:10.1128/JVI.00206-16 27009958 PMC4934756

[54] Isken O, Pham MT, Schwanke H, Schlotthauer F, Bartenschlager R, Tautz N. 2022. Characterization of a multipurpose NS3 surface patch coordinating HCV replicase assembly and virion morphogenesis. PLoS Pathog 18:e1010895. doi:10.1371/journal.ppat.1010895 36215335 PMC9616216

[55] Rinck G, Birghan C, Harada T, Meyers G, Thiel HJ, Tautz N. 2001. A cellular J-domain protein modulates polyprotein processing and cytopathogenicity of a pestivirus. J Virol 75:9470–9482. doi:10.1128/JVI.75.19.9470-9482.2001 11533209 PMC114514

[56] Venter PA, Krishna NK, Schneemann A. 2005. Capsid protein synthesis from replicating RNA directs specific packaging of the genome of a multipartite, positive-strand RNA virus. J Virol 79:6239–6248. doi:10.1128/JVI.79.10.6239-6248.2005 15858008 PMC1091714

[57] Venter PA, Schneemann A. 2007. Assembly of two independent populations of flock house virus particles with distinct RNA packaging characteristics in the same cell. J Virol 81:613–619. doi:10.1128/JVI.01668-06 17079301 PMC1797473

[58] Ansari IH, Chen LM, Liang D, Gil LH, Zhong W, Donis RO. 2004. Involvement of a bovine viral diarrhea virus NS5B locus in virion assembly. J Virol 78:9612–9623. doi:10.1128/JVI.78.18.9612-9623.2004 15331694 PMC515013

[59] Khromykh AA, Varnavski AN, Sedlak PL, Westaway EG. 2001. Coupling between replication and packaging of flavivirus RNA: evidence derived from the use of DNA-based full-length cDNA clones of Kunjin virus. J Virol 75:4633–4640. doi:10.1128/JVI.75.10.4633-4640.2001 11312333 PMC114216

[60] Fellenberg J, Dubrau D, Isken O, Tautz N. 2023. Packaging defects in pestiviral NS4A can be compensated by mutations in NS2 and NS3. J Virol 97:e0057223. doi:10.1128/jvi.00572-23 37695056 PMC10537661

