# Supplementary material for: Essential role of cis-encoded mature NS3 in the genome packaging of classical swine fever virus
Source: J Virol. 2024 Dec 26;99(2):e01209-24. doi: 10.1128/jvi.01209-24 (PMC11852850; doi:10.1128/jvi.01209-24)
Supplement: Supplemental legends — Legends for Movies S1 to S3. [file jvi.01209-24-s0001.docx]

**Legends for Movie S1, S2, and S3**

**Movies S1-S3.** Live-cell imaging. Imaging was performed using an EVOS M5000 system (Thermo Fisher) equipped with a Texas Red LED light cube. Naïve cells were seeded in a cell culture chamber on a glass slide, infected with 100 µl of supernatants from the transfected cells, and checked at 12h p.i. for fluorescence signals. Bright field and red fluorescence images were taken every 15 minutes until the cells were overgrown and dead. The resulting images were overlayed and compiled into time-lapse movies (3 frames per second).

**Movie S1:** Infection with supernatant of wtCSFV/CSFV-DI-mCherry. A DI-infected single cell appears in the center of the image and begins to detach. The infection started to spread to neighboring cells after approximately 24 hours., while additional infected cells appeared throughout the monolayer, with further spread observed after 35 hours and long-distance spread after approximately 60 hours. After about 72 hours, the entire monolayer showed signs of disruption and almost all cells were infected with the cytopathogenic DIs.

**Movie S2:** Infection with supernatant of wtCSFV/CSFV-DI-N_2177_Y-mCherry. Strong signals from a detached Doublet were visible 12 hours after infection and a first contact infection was already observed after 24 hours. However, the subsequent infection process in the monolayer was significantly delayed. Clear signals in neighboring cells and the formation of plaques were only visible after about 57h, and the formation of progeny plaques began at 80h p.i., when the infection process had already come to a halt due to cell overgrowth.

**Movie S3:** Infection with supernatant of CSFV-Δcore-N_2177_Y/CSFV-DI-N_2177_Y-mCherry. Weak mCherry signals appeared in a single cell, which subsequently detached from the plate surface and was lifted out of the monolayer. A prolonged gap phase, in which no neighboring cells were infected followed suggesting that CSFV-Δcore-N_2177_Y established infection more slowly before DI spread became possible. Only after about 57h p.i. stronger signals and plaque formation appeared in the neighboring cells. Although further spread, both near and far, began at approximately 80h p.i., the overall spread and plaque formation of the DI was limited in this combination.
